# Coding and Modulation for LMDS and Analysis of the LMDS Channel

**DOI:** 10.6028/jres.105.058

**Published:** 2000-10-01

**Authors:** Jan Erik Håkegård

**Affiliations:** National Institute of Standards and Technology, Gaithersburg, MD 20899-0001

**Keywords:** co-channel interference, Kaband channel, LMDS, modem design, nonlinear HPA, pragmatic TCM

## Abstract

Local Multipoint Distribution Service (LMDS) has the potential to become a viable alternative to coaxial cable, fiber and other wired and wireless technologies providing “last mile” communication services. A major obstacle, however, is the high equipment cost. While for example cable modems supporting two-way services are available for $200 to $300, LMDS modem providing similar services will cost over $1000. The major cost driver of LMDS equipment is the radio frequency (RF) unit, as Ka-band technology still is quite expensive. The modem design must minimize the overall architecture cost, and low-cost modems requiring an expensive architecture should not be used. The channel characteristics of LMDS systems are very different from those of fiber, coaxial cable, and lower frequency wireless links, major channel impairments being non-linear high power amplifier (HPA), high phase noise and high co-channel interference. Modems should therefore be developed specifically for LMDS systems. This report deals with the choice of coding and modulation schemes, the LMDS channel, and how the channel impairments should be overcome by digital signal processing algorithms.

## 1. Introduction

LMDS (Local Multipoint Distribution Services) is the American name for terrestrial point-to-multipoint (PMP) broadband communications systems transmitting on Ka-band (~30 GHz). In Canada these systems are referred to as LMCS (Local Multipoint Communications System), which is in fact more appropriate as two-way services are offered. In Europe similar systems go under the name MVDS (Multipoint Video Distribution Services), which is even less appropriate than LMDS, as audio and data as well as video are transmitted. Recently, the notation LMDS seems to have become more widely used, even in Europe.

Different frequency bands are allocated to LMDS systems in different countries. In the United States, the 24 GHz and 38 GHz bands, as well as the so-called U.S. LMDS spectrum of 1.3 GHz between 27.5 and 31.3 GHz, are used for PMP services. In Europe, the band between 40.5 GHz and 42.5 GHz is often used, but some countries have allocated other portions of the frequency band to LMDS type systems. Often, PMP systems transmitting on frequencies above 10 GHz are called LMDS systems, while systems transmitting on frequencies below 10 GHz are called MMDS (Multichannel Multi-point Distribution Services or Microwave Multipoint Distribution Services).

LMDS systems have been distributing analog TV signals in several places around the world since the beginning of the 1990s. The U.S. LMDS auction in 1998, where a large number of LMDS licenses were sold to a number of companies, triggered efforts to develop a new generation of LMDS systems to compete with wireline technologies like fiber, coaxial cable and xDSL. New LMDS systems must be able to handle a multitude of services, each with its data rate and requirement of quality of services (QoS). The traffic types may include voice, video, data services (T1/T3), TCP/IP traffic, and even full ATM switched services with both permanent and switched virtual circuits. Furthermore, the system must be flexible and adaptive, allowing dynamic changes in the transmitting and receiving traffic. To avoid the use of fixed channels the system must dynamically increase and decrease the data rate of open channels as the data requirements increase and decrease. The dynamic must be within the requirements and constraints of the overall system. For this new generation of LMDS systems to compete with other technologies, the operators need to find out which markets that are most likely to embrace LMDS as a “last mile” technology, and which services different market segments should be offered. The cost of equipment, and especially customer premise equipment (CPE), is another important factor, as Ka-band technology is still rather expensive.

This report is organized as follows. In Sec. 2, the LMDS architecture and network design are briefly described, together with future LMDS markets and services as industry leaders see them today. One strategy to reduce the component cost is to develop standards. Good standards that are complied to by the industry may reduce component cost through large scale production. In Sec. 3, LMDS specifications by DAVIC and ETSI concerning the physical interface are described. These standards are developed mainly for the first generation of LMDS systems, but are nevertheless of interest as many people in the LMDS industry prefer to reuse the same solutions, even though they are not optimal for the new generation of LMDS systems. Efforts are underway by the IEEE 802 LAN/MAN Standards Committee (IEEE 802.16) to develop an LMDS standard that is optimal for the new generation of LMDS systems. In Sec. 4, an alternative coding and modulation scheme is proposed. This scheme is not included in the IEEE 802.16 standard. The LMDS channel is very different from twisted pair, fiber and coaxial cable, and even from the MMDS channel. In Sec. 5, the main channel impairments are covered and approaches to overcome them are suggested.

## 2. LMDS Architecture and System Design

### 2.1 Introduction

In the United States, a total of 1.3 GHz of spectrum between 27.5 GHz and 31.3 GHz was auctioned out in February and March 1998 to be used for local distribution of voice, video and data. This is known as the U.S. LMDS spectrum. Other bands are also allocated for point-to-multipoint (PMP) systems at 24 GHz and 39 GHz. In Europe the band from 40.5 GHz to 42.5 GHz is generally used, but there are some differences between countries. For instance, in Germany the 24 GHz band is allocated for PMP services, while some other countries are using the 28 GHz band. Korea and Japan use frequencies from 22 GHz to 28 GHz.

### 2.2 System Architecture

**Fig. 1:**
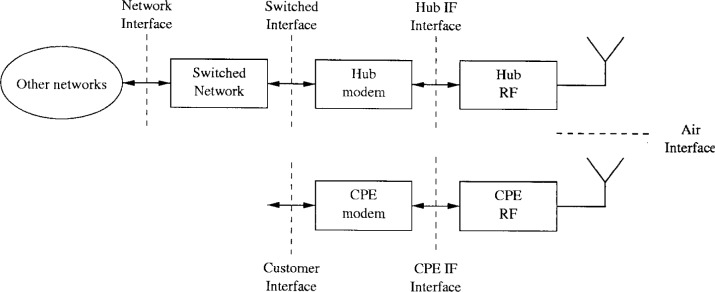
Schematic system block diagram of the physical layer of an LMDS system.

The four major elements of the LMDS network architecture are the base station equipment, RF equipment, customer premise equipment (CPE) and network management system. Fig. 1 illustrates the main sub-systems of the communication chain, and the interfaces connecting them.

The base station equipment typically consists of a network interface and a modem unit. The base station is the gateway between the wireless and wireline networks, and an ATM switch typically provides the connectivity. The modem functions include multiplexing, randomization, encoding and modulation for downstream data, and the inverse operations for upstream data. The modem connects to the radio frequency (RF) equipment through the intermediate frequency (IF) interface. The IF level is typically between 950 MHz and 2150 MHz.

#### 2.2.1 RF Equipment

The RF equipment consists of up/down conversion chains, a high power amplifier (HPA), a low noise amplifier (LNA) and filters. The high frequency band leads to significant line attenuation, so the RF elements are located close to the antenna. Multiple carriers on the same transceiver maximize throughput and reduce cost and complexity.

#### 2.2.2 Customer Premise Equipment (CPE)

The CPE contains an out-door unit (ODU) and an in-door unit (IDU). The ODU includes a 25 cm to 30 cm antenna and the RF unit. The CPE IF interface connects the ODU to the IDU. The CPE modem structures the data to emulate standard interfaces such as T1 or E1.

#### 2.2.3 Network Management System

The network management system (NMS) monitors the health and performance of the LMDS system by means of the agent applications and a management application. The NMS covers the operations, administration, maintenance and provisioning of the network. The agent applications are management software that resides in all elements of the network. The management application is a single application operated using a graphical user interface. It allows an operator to view the entire access network, and to optimize its performance.

### 2.3 Network Design

**Fig. 2:**
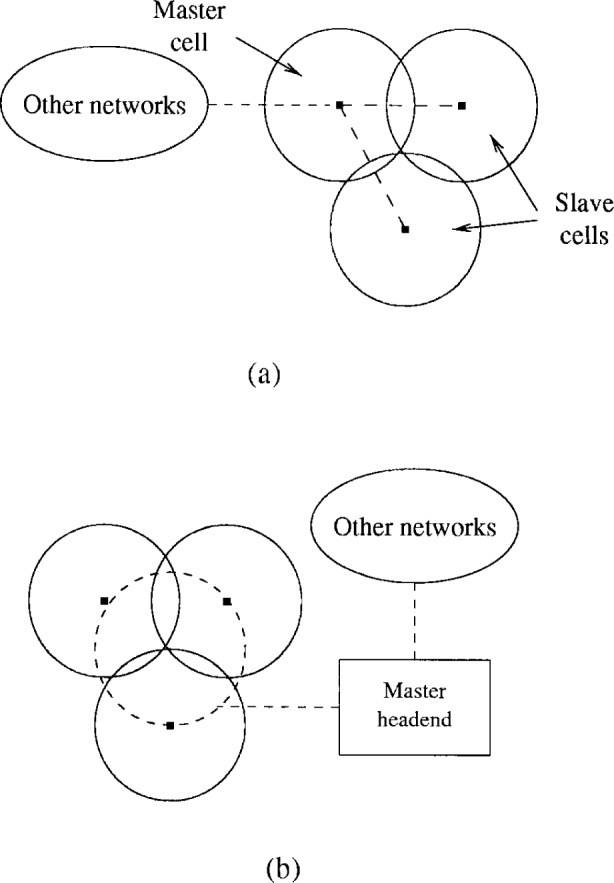
Cellularization of LMDS system.

Virtually everything represents an obstacle for signals at 30 GHz. The high free space loss and the line-of-sight constraint characterizing LMDS networks limit the area covered by one hub. Fig. 2a shows how a master cell can be connected to several slave cells, in order to cover a larger area. An alternative strategy is to interconnect the 3 hubs and a separate master headend by a wireless or fiber loop as shown in Fig. 2b. Both fully wireline and hybrid wireless/wireline solutions can be envisioned. The choice will depend on the current infrastructure, and on the available frequency resources. A system integrator with abundant spectrum may connect the hubs by wireless links, whereas an integrator with little spectrum to spare probably will use fiber.

The size of the circular cells typically depends on the system gain of the RF equipment, the transmit and receive antennas, the rain region in which the LMDS system is located, and the received signal-to-noise ratio required for acceptable system availability. Generally the cell radius will be between 2 km and 8 km without obstructions. In practical systems obstructions such as, buildings, vegetation and terrain, will block the signal in some directions. The shape of the cells will therefore be irregular, and even have “holes” within the cell boundaries where the signal from the hub can not be received. Careful coverage planning is necessary to maximize the availability throughout an area, and software packages using digital maps are being developed to facilitate this job. In some cases small active or passive repeaters within the cell can be used to reach areas lacking line-of-sight to the hub. In other cases large structures can be used as reflectors. The loss due to reflection will depend on the roughness of the surface. A third possibility is to have heavily overlapped cells. This solution also allows smart systems to use alternate transmission routes in the event of degraded capability in any one cell, as a portion of the customers may receive the signal from several hubs.

**Fig. 3:**
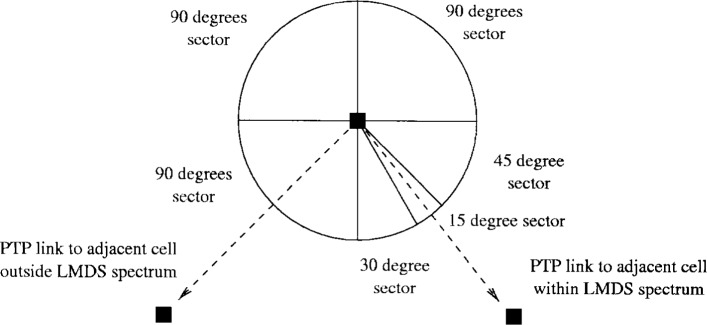
Sectorization of LMDS systems.

Although the LMDS spectrum is large compared to frequency bands designated to point-to-point (PTP) and PMP at lower frequencies, sectorization is used to increase the capacity. In Fig. 3 an example of how a cell can be divided into several sectors is illustrated. Increasing the number of sectors adds cost to the hub since the transceivers have to be replicated for each sector. It is possible to increase the number of sectors for the uplink only, relaxing the constraints on the CPE transmit power and increasing the uplink capacity. An other advantage of this approach is that a narrow beam receiver antenna reduces the effect of multipath propagation and hence inter symbol interference (ISI). An undesired effect of both cellularization and sectorization is co-channel interference (CCI). The effects of ISI and CCI on the system performance are covered in Secs. 5.5.2 and 5.5.3.

### 2.4 Markets and Services

Several market segments can be distinguished. LMDS operators will primarily target large businesses, and the services they offer will be trunked telephony, private data circuits, remote access and LAN interworking. These customers will be small in number, but well served by a large-bandwidth dedicated PTP link integrating a number of services. They will normally have internal wired networks with staff to maintain it. Line-of-sight will generally not be a problem as the customer antenna in most cases may be placed well above the ground and surrounding obstacles.

Medium to small businesses constitute another market segment. For many of these, a dedicated private link may not be a competitive alternative to other technologies. Often these businesses do not have staff or resources to maintain an internal network, and the LMDS operator must support parts of it. If a number of such businesses are geographically concentrated, they may be well served by an LMDS system with frequency division multiple access (FDMA) return links and a shared forward link.

Small offices/home offices (SOHO) is a potentially large and ever growing customer base. LMDS operators will however be reluctant to offer LMDS to this group for several reasons. The traffic pattern is rather sporadic with a large number of users sharing the spectrum, calling for time division multiple access (TDMA). The users are often located in leased site business parks or in single family dwellings, and it may be difficult to obtain line-of-sight between the base station antenna and the user antenna.

Residential users’ demand for bandwidth increases as the PC becomes more and more ubiquitous. For multi-dwelling units, LMDS systems may become an efficient alternative integrating voice, video and data services. Cable companies are, however, in the process of winning a big share of this market, and it is an open question whether LMDS CPEs will be able to compete in price with cable modems. Interoperable multimedia cable network system (MCNS) compliant modems are expected to be available for $200 to $300 by the end of 1999 [1]. For single family homes, it is doubtful if LMDS will ever become a serious competitor to wireline technologies.

In addition to providing “last mile” services, LMDS operators may provide PCS or cellular backhaul for other wireless carriers [2].

## 3. Standards and Specifications

Two organizations that have published standards for LMDS systems are DAVIC and ETSI. Version 1.0 of the DAVIC standards was finalized in 1996, the current and final Version 1.5 has been available on the Internet since the fall of 1999. The differences between Version 1.1 and Version 1.5 are minor, at least what concerns the physical interface. The ETSI LMDS standard was finalized in 1998, and is an extension of the DVB standard from 1997 for satellites using the 11 GHz to 12 GHz band, introducing interactivity into the system. In this section the parts of these standards concerning the physical interface are summarized.

In March 1999 the IEEE 802 LAN/MAN Standards Committee created the 802.16 working group on broadband wireless access. It focuses on standardizing US LMDS systems.

### 3.1 DAVIC

#### 3.1.1 About DAVIC

DAVIC (Digital Audio-Visual Council) was a non-profit association registered in Geneva, Switzerland. Among the 222 DAVIC members from 25 countries were manufacturers, service operators as well as a number of government agencies and research organizations. The purpose of DAVIC was to specify open interfaces and protocols that maximize interoperability across countries, applications and services. According to its statutes, DAVIC was closed in 1999 after 5 years of activity. The final release is version 1.5 (June 1999).

The DAVIC standard includes three wireless communications technologies: satellite communications, MMDS, and LMDS. Furthermore, broadband communications over copper, fiber and coaxial cable are included. DAVIC defines MMDS as terrestrial broadband communications at frequencies below 10 GHz and LMDS as terrestrial broadband communications above 10 GHz. The specifications contain 14 parts, but only Part 8: “Lower-Layer Protocols and Physical Interfaces” is covered here. The DAVIC specifications provide interfaces for both narrowband (PSTN, ISDN and PLMN) and broadband (SDH, SONET, PDH) core networks. Two frame structures are provided, one for MPEG-2 Transport Streams and the other for ATM-cell transfer.

#### 3.1.2 Downstream

The access scheme for downlink is TDM with frame length between 3 ms and 6 ms. The channel bandwidth is defined to be between 20 MHz and 40 MHz. The frames contain a number of time slots, which are divided into start slots and random access slots. To limit the amount of processing required by the CPE, it will not receive more than 7 % of the slots within a frame. This includes both broadcasting cells and cells directed specifically to the CPE. With a total downstream bit rate of 51 Mbit/s, this corresponds to 50 ATM cells or 14 MPEG2-TS packets per 6 ms frame. Each CPE can then receive up to about 3.5 Mbit/s. The hub is capable of transmitting on several channels simultaneously. However, the CPE may receive only one channel. It may switch to different frequency channels, but this switch may require time in the order of several time slots.

The frame structure is in accordance with MPEG2 Transport Stream. Below the frame structures carrying MPEG2-TS and ATM are briefly described.

**Fig. 4:**
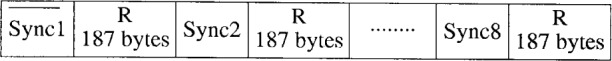
MPEG2-TS frame structure.

The MPEG2-TS MUX packet has 188 bytes, with one synchronization byte, three bytes of header containing service information, scrambling and control information, followed by 184 bytes of MPEG2 or auxiliary data. The packets are randomized using a pseudo random binary sequence (PRBS) of period 1503 bytes, corresponding to 8 packets (see Fig. 4). The sync bytes are not randomized. The first sync byte is complemented.

**Fig. 5:**
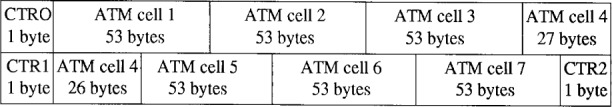
ATM frame structure.

The ATM packets contain 53 bytes. In order to fit ATM cells into 187 B packets, two-packet sequences are defined (see Fig. 5). Each two-packet sequence contains 7 ATM cells and 3 control bytes. In addition come two sync bytes to complete the 188 B MUX packets. The control bytes CTR0 and CTR1 indicate if a packet is the first or the second packet in the sequence. They also contain an error flag and stuffing bits. CTR2 will be defined for carriage of operation, administration and maintenance information (OAM).

**Fig. 6:**
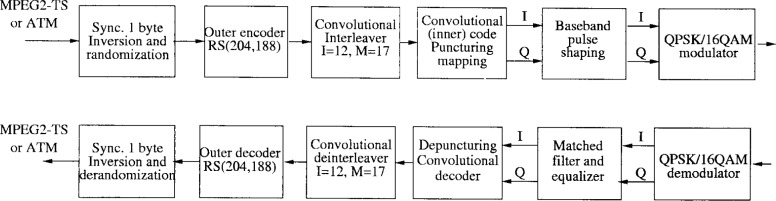
Block diagram for the transmitter and receiver for downstream LMDS.

In the specifications, the channel coding is divided into 5 parts: randomization, Reed-Solomon outer coding, convolutional interleaving, convolutional inner coding, and mapping. The transmitter and receiver block diagrams are illustrated in Fig. 6.

The randomization ensures adequate binary transition for clock recovery. At the start of every 8 transport packet, the sequence “100101010000000” is loaded into a linear feedback shift register (LFSR) with polynomial 1 + *x*^14^ + *x*^15^. Systematic shortened Reed-Solomon coding is applied. The encoder adds 16 parity bytes (RS(204,188)), and can consequently correct up to 8 erroneous bytes per packet. The code has the following polynomials:

- Code Generator Polynomial: *g*(*x*) = (*x* +*μ*^0^)(*x* + *μ*^1^) …(*x* + *μ*^15^), where *μ* = 02hex
- Field Generator Polynomial: *p*(*x*) = *x*^8^ + *x*^4^ + *x*^3^ + *x*^2^ + 1

The shortened RS code is obtained by appending 51 bytes, all set to zero, before the information bytes at the input of a (255,239) encoder. The 51 bytes are discarded after the coding procedure.

The conventional interleaver contains *I* = 12 branches, where the *i* th branch (*i* = 0,…, *I* −1) contains *i* FIFO shift registers. The shift registers have *M* = 17 cells, each cell containing 1 B. The delays at the output of the interleaver is then equal to *iM*. The sync bytes are always routed into the 0th branch corresponding to zero delay.

Convolutional coding is only applied with QPSK modulation. A number of punctured codes based on a rate 1/2 code are allowed. The punctured code rates are 2/3, 3/4, 5/6 and 7/8. The original rate 1/2 code has constraint length 7, and is characterized by the function generators:

- *g*_1_ = 171 oct
- *g*_2_ = 133 oct

**Fig. 7:**
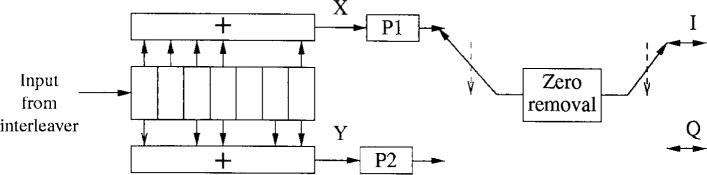
Block diagram over encoder with puncturing.

The punctured codes are defined in Table 1. The 0’s in the table correspond to input bits coming from the rate 1/2 convolutional encoder that are not transmitted. Fig. 7 shows the block diagram of the encoder. To illustrate how the puncturing works, suppose that the sequences {*x*_1_, *x*_2_, *x*_3_} and {*y*_1_, *y*_2_, *y*_3_} are the inputs of P1 and P2, and that the code with rate 3/4 is chosen. The outputs of P1 and P2 are then {*x*_1_, 0, *x*_3_} and {*y*_1_, *y*_2_, 0}. After the zero removal the sequence becomes {*x*_1_, *y*_1_, *y*_2_, *x*_3_}. The inphase (I) and quadrature (Q) channels sent to the mapper are then given by {*x*_1_, *y*_2_} and {*y*_1_, *x*_3_}, respectively. The receiver tries and checks frame lock acquisition to detect which coding rate is used.

**Fig. 8:**
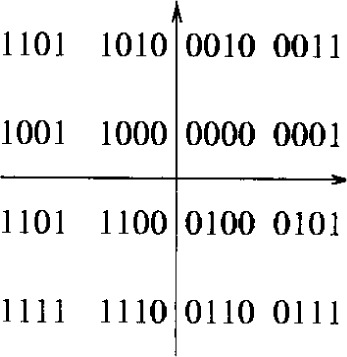
16QAM Constellation diagram with mapping.

The DAVIC specifications define grad A and B communication. Grad A includes only QPSK modulation, grad B includes both QPSK and 16QAM. A modulator must support at least one of the modulations (QPSK and 16QAM), while a demodulator must support grad A or B communication. For QPSK modulation, conventional Gray mapping is employed. For 16QAM, the bytes at the output of the interleaver are split in two in order to form 4-bit symbols. The constellation diagram with mapping is illustrated in Fig. 8. The I and Q channels at the output of the mapper are filtered using a square-root raised cosine filter with roll-off factor 0.2 or 0.35.

#### 3.1.3 Upstream

**Fig. 9:**
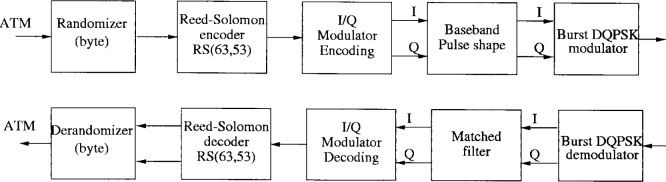
Block diagram for the transmitter and receiver for upstream LMDS.

The block diagrams of the transmitter and receiver are illustrated in Fig. 9.

The access scheme for the uplink is TDMA. The frame length is the same as for the downlink, between 3 ms and 6 ms The CPE adjusts timing of upstream packet transmissions to coincide with the upstream frame slot boundaries at the hub within a specified level of accuracy. Before net entry, the accuracy is determined by uncertainties about propagation delay, i.e., the cell radius. Net management allocates multiple contiguous time slots to form longer net entry slots such that TDMA packet collisions caused by CPE net entry transmissions are precluded. Synchronization pull-in is provided as the CPE adjusts the transmission time in response to feedback of synchronization error estimated by the hub. After net entry, the synchronization error shall be maintained less than one upstream symbol duration (the packet guard time is 4 symbols). The hub provides periodic feedback of synchronization error to maintain the accuracy.

**Fig. 10:**
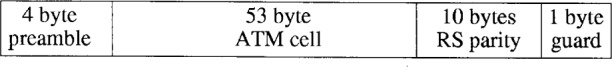
Upstream time slot structure.

The upstream packets contain 68 B (see Fig. 10). The upstream frame consists of *F* slots where *F* depends on the upstream modulation rate used. The *F* slots are partitioned into polling slots, contention slots and reserved time slots. The relative number of these types of slots is dynamic.

- Polling slots may only be utilized for poll response after receiving a poll request from the hub.
- Utilizing the contention slots may cause collisions, which may be resolved using one of many well-known algorithms such as random retransmission delay.
- Reserved time slots are reserved for one user only.

When a CPE attempts to enter the network, it acquires a downstream frequency channel and listens for the poll containing its full or partial serial number. If the CPE waits for 2 s without receiving the poll directed to it, it will acquire the next frequency and again listen for its serial number. This process repeats itself until the CPE finds a downstream frequency channel on which it is being polled. The CPE responds to the poll, and is calibrated, or time aligned. Contention or reserved time slots may then be allocated. The polling of the CPE is continued to maintain upstream transit signal calibration. The CPE is only capable to transmit at one channel at the time. However, it is capable of switching channels between time slots.

RS coding is applied to the randomized ATM cell. The encoder adds 10 parity bytes (RS(63,53)), and can correct up to 5 erroneous bytes per packets. The code has the following polynomials:

- Code Generator Polynomial: *g*(*x*) = (*x* + *μ*^0^)(*x* + *μ*^1^) …(*x* + *μ*^9^), where *μ* = 02h
- Field Generator Polynomial: *p*(*x*) = *x*^8^ + *x*^4^ + *x*^3^ + *x*^2^ + 1

The RS code is obtained by appending 192 zero symbols before the information bytes at the input of a (255,245) encoder. The 192 bytes are discarded after the coding procedure. The I and Q channels at the output of the encoder are filtered by a square-root raised cosine filter with roll-off 0.3. The modulation scheme is differential QPSK.

### 3.2 ETSI

#### 3.2.1 About ETSI

The European Telecommunications Standards Institute (ETSI) is a non-profit making organization whose mission is to determine and produce telecommunications. It is an open forum that unites 490 members from 34 countries, representing administrations, network operators, manufacturers, service providers, and users.

ETSI promotes the worldwide standardization process whenever possible. Its Work Program is based on, and coordinated with, the activities of international standardization bodies, mainly the ITU-T and the ITU-R.

ETSI consists of a General Assembly, a Board, a Technical Organization and a Secretariat. The central Secretariat of ETSI is located in Sophia Antipolis in Southern France. The Technical Organization produces and approves technical standards. It encompasses ETSI Projects (EPs), Technical Committees (TCs) and Special Committees. More than 3500 experts are at present working for ETSI in over 200 groups. Up to now, over 2800 ETSI deliverables have been published.

#### 3.2.2 The ETSI LMDS Specifications

The title of these specifications is “Digital Video Broadcasting (DVB); Local Multi-point Distribution Service” [4]. The DVB standards are generally developed for satellite broadcasting at 11/12 GHz [5]. The purpose of LMDS is to provide interactivity in the DVB environment.

ETSI distinguishes between the broadcast channel (BC) and the interaction channel (IC):

- The BC is unidirectional and broadband. It is used for video, audio and data.
- The IC is bi-directional, comprised by a forward interaction path and a return interaction path.
- The forward interaction path may be embedded into the BC. It is used to provide information and required communication from the service provider to the user.
- The return interaction path is used to make requests to the service provider or to answer questions.

These specifications are best suited for systems offering TV and Internet access to a large number of residentials, rather than systems serving a small number of large businesses needing fast two-way communications.

The interactive system is based either on OOB or IB signaling. Both systems can provide same QoS.

- OOB signaling: An additional forward interaction path, reserved for interactivity data and control information, is mandatory. Higher bit rate downstream data can be transmitted through a DVB-MS (DVB-Microwave Satellite) channel.
- IB signaling: The forward information path is embedded into the MPEG2-TS of a DVB-MS channel. It is however not mandatory to put a forward information path in all DVB-MS channels.

Downstream information is transmitted broadcast to all users. Address assignments allow the hub to send information to one particular user.

#### 3.2.3 Downstream OOB Transmission

OOB downstream transmission is divided into 2 MHz channels. For the interactive downstream OOB channel, a rate of 3.088 Mbit/s may be used. For the OOB broadcast channels, a transmit frame based on T1 type framing is used.

The Signaling Link Extended Superframe (SL-ESF) consists of 24 bit × 193 bit frames, a total of 4 632 bits. Each frame contains one overhead bit and 24 bytes of payload. The payload structure consists of 5 rows of 57 bytes each, 4 rows of 58 bytes each including 1 byte trailer, and 1 row of 59 bytes including a 2 bytes trailer. The trailer bytes are not used, and set to zero. The 57 bytes include 1 53 B ATM packet, 2 RS redundancy bytes, and 2 bytes containing slot configuration information for the upstream channel.

**Fig. 11:**
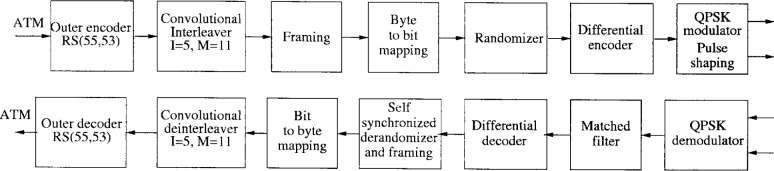
Block diagram for the transmitter and receiver for downstream out-of-band (OOB) transmission.

The block diagrams of the transmitter and receiver are illustrated in Fig. 11. The RS(55,53) encoder adds 2 parity bytes, which means that one erroneous byte per ATM cell can be corrected. The RS code has the polynomials:

- Code Generator Polynomial: *g*(*x*) = (*x* + *μ*^0^)(*x* + *μ*^1^), where *μ* = 02h
- Field Generator Polynomial: *p*(*x*) = *x*^8^ + *x*^4^ + *x*^3^ + *x*^2^ + 1

The convolutional interleaver is of size *I* = 5, *M* = 11. QPSK modulation with Gray mapping is used, and the roll-off factor is equal to 0.3.

#### 3.2.4 Downstream IB Channel

The downstream IB channel uses the standards defined for DVB for 11 GHz to 12 GHz satellite services [5]. The system is optimized for single carrier per transponder TDM, but it is able to handle multi-carrier FDM type applications. As a guideline, the data rate is set to multiples of 8 kbps. The frame structure is equal to the MPEG2-TS frame structure defined in the DAVIC specifications, containing 188 bytes.

**Fig. 12:**
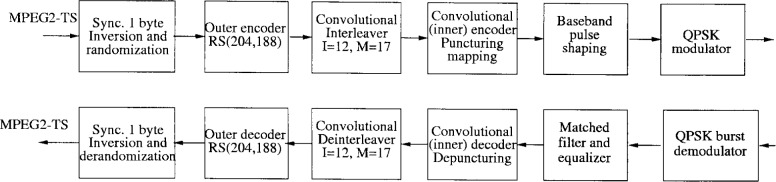
Block diagram for the transmitter and receiver for downstream in-band (IB) transmission.

The block diagram for downlink IB communications are illustrated in Fig. 12. The Reed-Solomon RS(204,188) shortened code is specified as outer code, which is the same as specified for downlink by DAVIC. With an input BER of about 7 × 10^−4^ or better with infinite byte interleaving, the decoder should provide QEF (quasi error free) output, i.e., BER of about 10^−10^ to 10^−11^. The convolutional interleaver is the same as that specified for the downstream path in the DAVIC specifications (*I* = 12, *M* = 17), and so is the inner coding. The inner decoder should operate at an input equivalent “hard decision” BER between 10^−1^ and 10^−2^, and should produce an output BER of about 2 × 10^−4^ or lower.

The roll-off factor of the square root raised cosine filter is 0.35, which differs slightly from the DAVIC specifications, where both 0.2 and 0.35 are allowed.

The use of a FIR digital filter can provide equalization of the channel linear distortions. This filter is not specified, but will depend on the actual transmission channel. The modulation scheme is conventional Gray-coded QPSK.

#### 3.2.5 Upstream

The access scheme for the uplink is FDM/TDMA. A slotting method is used to increase the throughput. The time reference for the slot location is received via the downstream channel. There are several access modes:

- reserved slots with fixed rate (a given bit rate requested by the user is transmitted until the base station stops the connection on the user’s demand)
- reserved slots with dynamic reservation (base station provides a finite amount of slots to a specific user)
- contention based slots (accessible for every user, collisions possible)
- ranging slots (used to measure and adjust time delay and power)

These access modes are dynamically shared among time slots. In contention access mode, a positive acknowledgment is sent back to the CPE for each successfully received ATM packet. If a collision occurs, the CPE will retransmit using a procedure to be defined.

Upstream communications are divided into 2 MHz channels. The framing consists of packets of 512 bits, which are sent in bursty mode. The upstream slot rate is 6000 slots/s.

When some message slots are unused, a user can be assigned multiple slots for increased throughput. The different access modes can be mixed on a single carrier, or a carrier can be assigned to one specific service.

When a user enters the network, he follows a so-called Sign-On Procedure. The base station sends periodically a sign-on request to allow a CPE to indicate its presence in the network. The CPE responds with a Sign-On Response Message. To adjust the time offset and the power, a Range and Power Calibration Message is sent from the base station. When the CPE is calibrated in time within a window of 1.5 symbols and in power within a window of 1.5 dB from the optimal values, an Initiation Complete message is sent. Once a CPE has completed the Calibration State, it enters the Connection State.

**Fig. 14:**
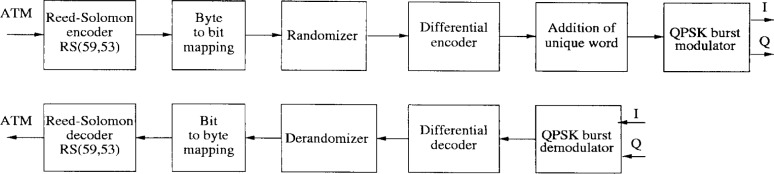
Block diagram for the transmitter and receiver for upstream transmission.

The block diagram of the transmitter and receiver are illustrated in Fig. 14. RS(59,53) coding is performed on each ATM cell. The code can correct 3 erroneous bytes per packet. The code is given by the following generating polynomials:

- Code Generator Polynomial: *g*(*x*) = (*x* + *μ*^0^)(*x* + *μ*^1^) …(*x* + *μ*^5^), where *μ* = 02h
- Field Generator Polynomial: *p*(*x*) = *x*^8^ + *x*^4^ + *x*^3^ + *x*^2^ + 1

Differential QPSK is used, and the roll-off factor is 0.3 as for downlink OOB.

In Table 2 some IF interface parameters of the DAVIC and ETSI specifications are summarized.

### 3.3 IEEE 802

In August 1998 N-WEST (National Wireless Electronic Systems Testbed), an initiative from two agencies of the U.S. Department of Commerce: the National Institute of Standards and Technology (NIST) and the National Telecommunications and Information Administration (NTIA), was kicked off. One of the goals of N-WEST was to promote standardization of LMDS systems. Five months later, the effort had resulted in a study group (“802.N-WEST”) under the IEEE 802 LAN/MAN Standards Committee. In March 1999, the IEEE created the 802.16 working group on broadband wireless access. The standard will specify the physical layer (PHY) and media access control (MAC) layer of the air interface of interoperable fixed PMP broadband wireless access systems. It will concentrate on systems operating in the vicinity of 30 GHz, i.e., in the US LMDS band, but be applicable to systems operating on frequencies between 10 GHz and 66 GHz. More than 100 companies, including manufacturers, system integrators and operators are supporting N-WEST, and the number is increasing.

It is believed that the work of this standardization group is crucial for the future of LMDS, at least in the United States. It will be the first LMDS standard to match the types of services and markets relevant for the LMDS systems of the next five to ten years. Hence, the timing of the work is very good. As a number of market leading companies are involved in the standardization work, it also has the potential to be widely recognized and complied to by the industry. However, many different interests are involved, and the standardization group has to overcome these differences in order to become a success.

### 3.4 BER Performance for Transmission Over a Flat Additive Gaussian Channel

One of the goals of this project was to simulate transmission of data over an LMDS link. A simulation model was built of the upstream and downstream transmission chains as specified by DAVIC and ETSI. The simulation tools used were MATLAB and Simulink. In order to verify the simulation models, the performances of the links were calculated theoretically for transmissions over the additive white Gaussian noise (AWGN) channels. The results apply to the ideal case of no interference, perfect timing and synchronization, and ideal RF components. When it is established that the simulation results coincide with the theoretical results, the effects of other channel impairments like ISI, CCI and non-linearities can easily be evaluated by simulation.

#### 3.4.1 Theoretical Analysis

Some additional assumptions have been made in the computation of the BER performances. In the convolutional decoder hard decision is used. The output BER can then be derived from the input BER from the table on page 402 in Clark and Cain [6]. In the case of Reed-Solomon coding, the interleaving is assumed to be perfect. The RS code performance is then derived from the combinatorial formulas given in the literature.

The modulation formats used in the DAVIC and ETSI standards are QPSK, differential QPSK and 16QAM. The derivation of the expressions below are taken from ref. [7].

An approximation of the BER with QPSK modulation is given by:

$$P_{\text{QPSK}}=\frac{1}{2}\text{erfc}\left(\sqrt{\gamma b}\right)$$ (1)

where *γ*_b_ is the SNR per bit and erfc(*x*) is the complementary error function:

$$\text{erfc}(x)=\frac{2}{\sqrt{\pi }}\int_{x}^{\infty }e^{-\frac{t}{2}}\text{dt}.$$ (2)

Gray coding is used, and Eq. (1) makes the approximation that one symbol error equals one bit error.

For differential QPSK the expression of the performance is slightly more complex.

$$P_{\text{DQPSK}}=e^{-\frac{a^{2}+b^{2}}{2}}\left(\sum_{k=0}^{\infty }{\left(\frac{a}{b}\right)}^{k}I_{k}(ab)-\frac{1}{2}I_{0}(ab)\right)$$ (3)

where:

$$a=\sqrt{\gamma_{b}(2-\sqrt{2})},\;b=\sqrt{\gamma_{b}(2+\sqrt{2})}.$$ (4)

When Gray mapping is used with 16QAM, two nearest neighbors in the signal constellation only differ in one bit (see Fig. 8). The BER can then be approximated as 1/4 of the symbol error rate, and expressed as:

$$P_{16\text{QAM}}\approx \frac{3}{8}\text{erfc}\left(\sqrt{\frac{2\gamma_{b}}{5}}\right)-\frac{9}{64}{\text{erfc}}^{2}\left(\sqrt{\frac{2\gamma_{b}}{5}}\right).$$ (5)

A rate 1/2 convolutional code with constraint length *K* = 7 is used. The pairwise error probability (PEP) is used to calculate the union bound of the BER. The PEP is given by:

$$P_{2}(d)=\{\begin{array}{ll} \sum_{k=\frac{d+1}{2}}^{d}\left(\begin{array}{c} d\\ k \end{array}\right)p^{k}{\left(1-p\right)}^{d-k} & \text{if}\;d\;\text{is odd}\\ \sum_{k=\frac{d}{2}+1}^{d}\left(\begin{array}{c} d\\ k \end{array}\right)p^{k}{\left(1-p\right)}^{d-k}+\frac{1}{2}\left(\begin{array}{c} d\\ \frac{1}{2}d \end{array}\right)p^{\frac{d}{2}}{\left(1-p\right)}^{\frac{d}{2}} & \text{if}\;d\;\text{is even} \end{array}$$ (6)

where *p* is the probability of a bit error at the input of the decoder. The BER at the output of the decoder is upper bounded by:

$$P_{\text{cc}}\leq \frac{1}{2}\sum_{d=d_{\text{free}}}^{\infty }w_{d}P_{2}(d)$$ (7)

where *d*_free_ is the free distance of the code. The weights *w_d_* can be found by a program searching through the trellis for all possible error paths. For some of the most used codes, however, the weights can be found in the literature. As short error paths occur more often than long error paths, the summation in Eq. (7) may be limited to a finite number of terms. In Table 3, the first 5 weights of the code used are listed. Only taking into account these terms, the expression is not longer a true union bound, and is sometimes called a truncated union bound (TUB). The error due to the truncation is very small.

There are several expressions available in the literature giving the performances for Reed-Solomon codes. One of them is an upper bound derived in [7]:

$$P_{\text{RS}}\leq \frac{2^{k-1}}{N(2^{k}-1)}\sum_{i=t+1}^{N}i\left(\begin{array}{c} N\\ i \end{array}\right)P_{S,\text{in}}^{i}{\left(1-P_{S,\text{in}}\right)}^{N-i}$$ (8)

where *t* = ⎿(*N K*)/2⏌ is the number of errors per packet the code is capable of correcting. The input symbol error probability is related to the input bit error probability in a straight forward way:

$$P_{s,\text{in}}=1-{\left(1-P_{b,\text{in}}\right)}^{k}$$ (9)

where *k* is the number of bits per symbol. The bound in Eq. (8) is not very accurate for high BERs, and a better approximation of the performance is to choose output BER to be the minimum of the input and the output BERs.

#### 3.4.2 Diagrams

**Fig. 16:**
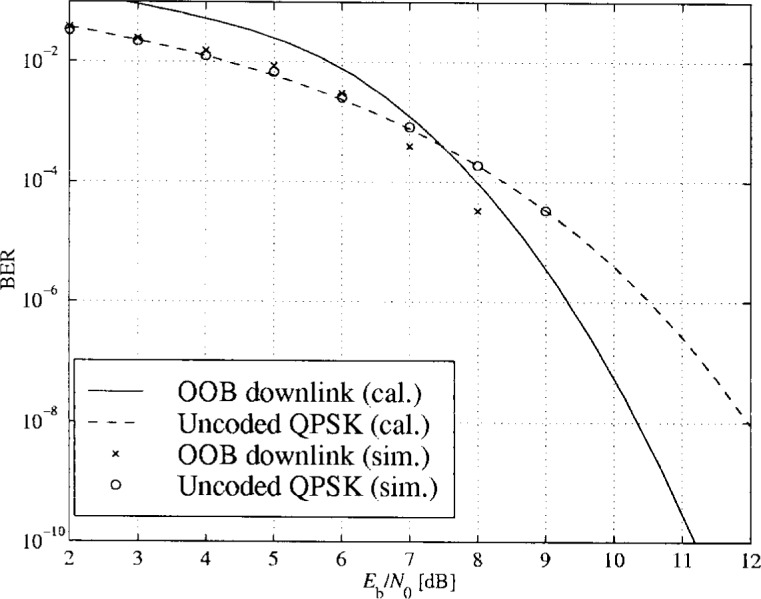
Performance for ETSI OOB downlink (RS(55,53) coding, QPSK modulation).

**Fig. 17:**
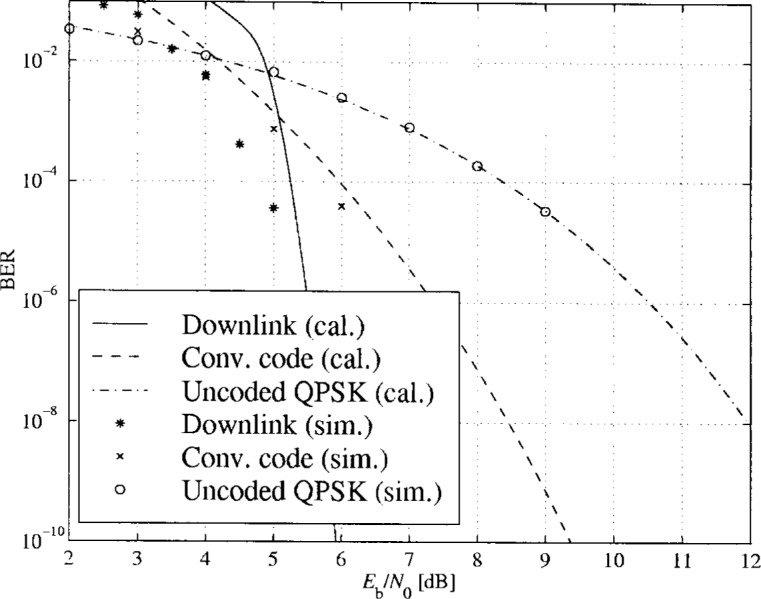
Performance for DAVIC and ETSI IB downlink with no puncturing (RS(204,188) outer code, rate 1/2 convolutional inner code, QPSK modulation).

**Fig. 18:**
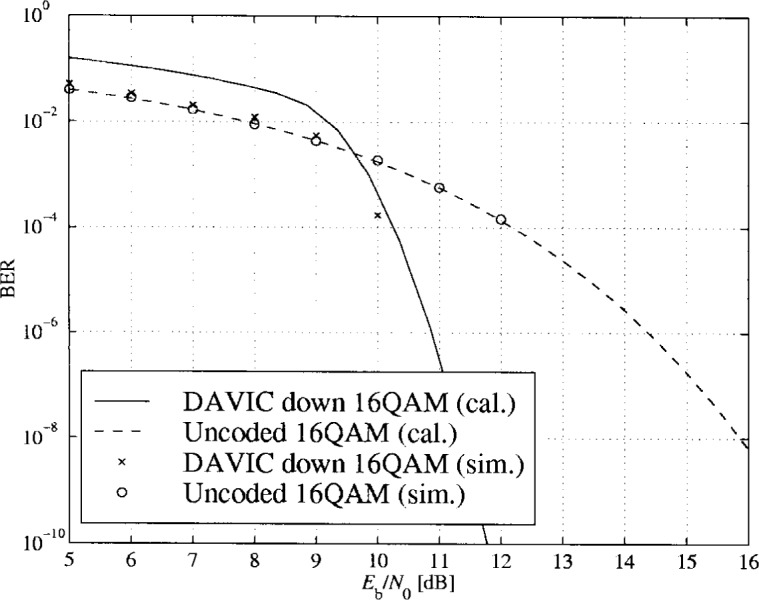
Performance for DAVIC downlink with 16QAM and RS(204,188) coding.

In Figs. 16 to 18 the BER performances of the uplinks and downlinks of the DAVIC and ETSI specifications are shown. Both calculated and simulated values are shown, the lines corresponding to calculated BER values and the markers to simulated BER.

## 4. A Fully Rotational Invariant Coding and Modulation Scheme

### 4.1 Introduction

The punctured convolutional codes used by the ETSI and DAVIC LMDS specifications were first employed by Linkabit Corp.^1^, and appeared in the literature in 1979 [8] for rate 2/3 and rate 3/4, and for arbitrary rate *m*/(*m* + 1) in 1984 [9]. Their main advantage is the unifying implementation of the encoder/decoder with different code rates. The different code rates require that the modulator operates at different data rates and hence different bandwidths. There is another approach that has the advantage of unified implementation with different data rates, but that does not require different bandwidths. The approach is called Pragmatic trellis coded modulation (PTCM), and was proposed by Viterbi et al. in 1989 [10]. As in traditional TCM, the different coding rates are obtained by choosing different modulation schemes. In wireless multirate communication systems, where the number of users may vary dynamically, as do their required data rates, this feature adds flexibility to the system.

**Fig. 19:**
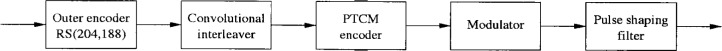
Block diagram of the transmitter.

In this section an alternative coding and modulation scheme is proposed. The transmitter block diagram is illustrated in Fig. 19. The same outer code and interleaver is used as in the downlink specifications of DAVIC and ETSI (IB). Instead of using a punctured convolutional code as inner code, this scheme uses PTCM.

In the early stages of the development of the IEEE 802.16 standard, PTCM was considered as an alternative modulation scheme. It was however dropped, and will not be supported by the final IEEE 802.16 standard.

### 4.2 Pragmatic TCM (PTCM)

The optimum TCM codes for the additive white Gaussian noise (AWGN) channel are the so-called Ungerboeck codes [11]. They are based on the set partition principle, where an *M*-ary constellation is successively partitioned into 2, 4, 8,…subsets with size *M*/2, *M*/4, *M*/8,…, having progressively larger minimum Euclidean distances. To achieve the optimum code designs in a multirate environment requires implementing different encoders and decoders for each code rate. The PTCM codes constitute a suboptimum solution allowing the implementation of several coding rates using the same encoder. Decoders with identical trellis connectivities make it very easy to implement a common decoder for all the data rates.

**Fig. 20:**
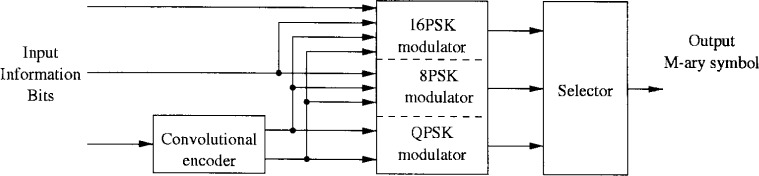
Pragmatic TCM encoder for *M*PSK signal.

**Fig. 21:**
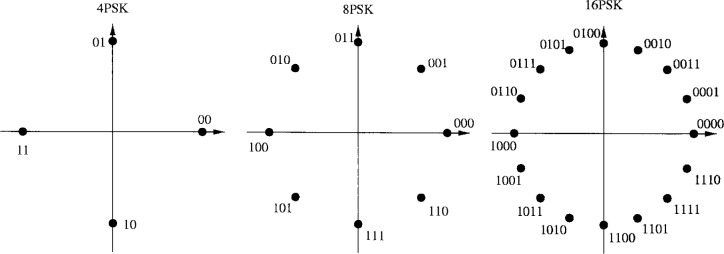
Constellation diagrams and mapping with *M*PSK modulation.

**Fig. 22:**
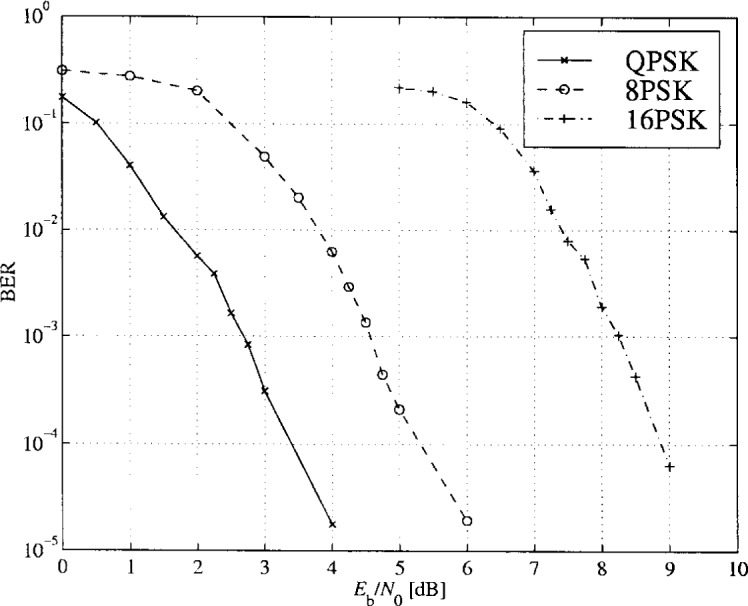
Simulated BER with PTCM coding and *M*PSK modulation.

**Fig. 23:**
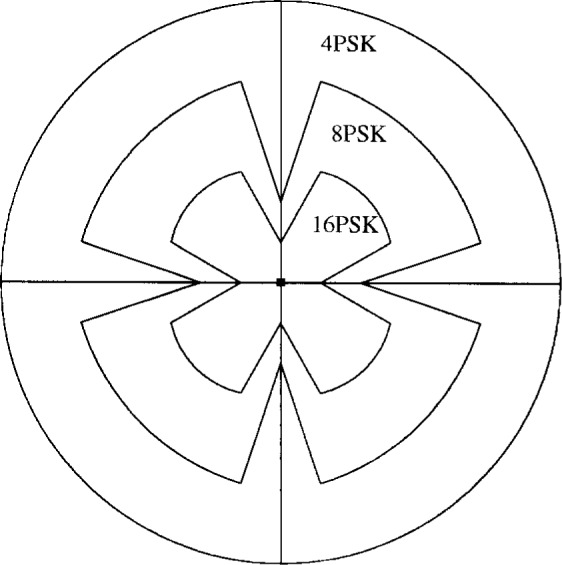
Example of how a cell may be divided into zones defined by highest modulation scheme permitted to obtain required signal quality and system availability to all users.

The PTCM encoder is illustrated in Fig. 20 4 PSK, 8 PSK, and 16 PSK modulation, and the mapping is depicted in Fig. 21. The same convolutional code of rate 1/2 and constraint length 7 is used as in the ETSI and DAVIC specifications. This code has been around since the early 1970s [12], and a VLSI implementation of it has been available for over 10 years [13]. For QPSK, the PTCM encoder works identically to the convolutional encoder with rate 1/2. The two outputs of the encoder define one out of 4 symbols in the constellation using Gray mapping. For 8PSK, these symbols are uniformly located in either the right or left half-plane, with the remaining uncoded bit defining which half-plane to choose. For 16 PSK, the four symbols are located in one of the four quadrants. The two uncoded bits are used to choose the quadrant. This principle can be generalized to arbitrary *M*PSK with *M* = 2*^m^*^+1^. In each case the lowest-ordered of the *m* input bits is fed to the encoder whose output bits define one of four phases within a sector defined by the angle (2π/2*^m^*^−1^) radians using Gray mapping. The remaining *m* − 1 bits select the sector lexicographically [10]. The performances of PTCM codes are very close to those of the Ungerboeck codes. With 16PSK modulation, the performance is almost the same for all BERs of interest. With 8PSK modulation, the performances are close to identical for BERs down to 10^−4^. For lower BERs the coding gain of the PTCM is lower than that of the Ungerboeck code. At a BER of 10^−5^ the difference is about 0.3 dB, which is still small [10]. In Fig. 22, the simulated BER performance of the PTCM codes with *M*PSK is shown. When 8PSK is used, the *E*_b_/*N*_0_ requirements, where *E*_b_ denotes the energy per *uncoded* bit to achieve a BER of 10^−4^, is about 2 dB higher than that of QPSK. For 16PSK it is about 6 dB higher. For subscribers located close to the edge of the cell, and in locations where the interference level is high, it will be impossible to obtain reliable communications using 16PSK and even 8PSK. For customers located close to a hub, however, the *C*/(*I* + *N*) (*carrier-to-interference-plus-noise ratio*) is large enough to permit 16PSK modulation. A cell can be divided into zones defined by the highest ordered modulation allowed to ensure required signal quality and system availability. In that way the total throughput is increased. In Fig. 23, an example on how a cell can be divided into areas appropriate for different modulation schemes is illustrated.

**Fig. 24:**
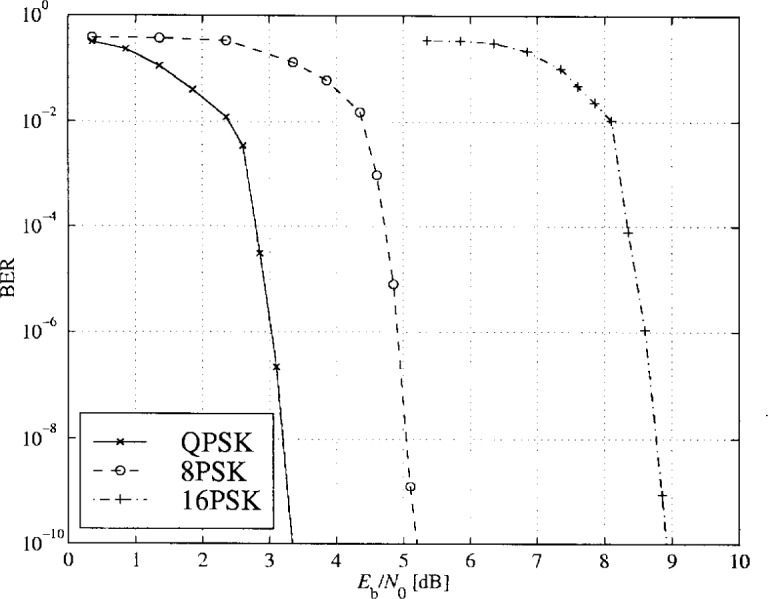
Calculated BER with RS(204,188) outer coding, PTCM inner coding and *M*PSK modulation.

To achieve quasi error free (QEF) transmission, i.e., a bit error rate about 10^−10^ 10^−11^ or one error per hour, PTCM is used in combination with convolutional interleaving and outer Reed-Solomon (RS) coding. The size of the interleaver must be matched to the characteristics of the channel and to the coding and modulation scheme. The size of the RS code must be adapted to the packet length. In Fig. 24, the BER performance is calculated using the simulation results with PTCM coding only, and Eq. (8), which is an upper bound.

The concept of PTCM can be extended to pragmatic punctured TCM (P^2^TCM) [19], for which punctured versions of the rate 1/2 convolutional code are used instead of the rate 1/2 code. This approach is reported to give more efficient codes than the non-punctured PTCM codes [19].

### 4.3 Modulation Techniques

To maximize the capacity of the communication system, QPSK with a spectral efficiency of only 2 (bit/s)/Hz is becoming less attractive. However, there are several reasons why the spectral efficiency should not be chosen too high either. One obvious reason is the extra power needed to achieve the same performance as systems with lower spectral efficiency. The cost of the high power amplifier (HPA) increases with the transmitted power. The HPA also introduces nonlinear distortion that may do severe damage for multi amplitude constellations with large envelope fluctuations. Moreover, a high order modulation scheme makes the system more sensible to other channel impairments like CCI. The result is that the requirements on the system architecture become very strict, and hence, the cost of the equipment become high.

**Fig. 25:**
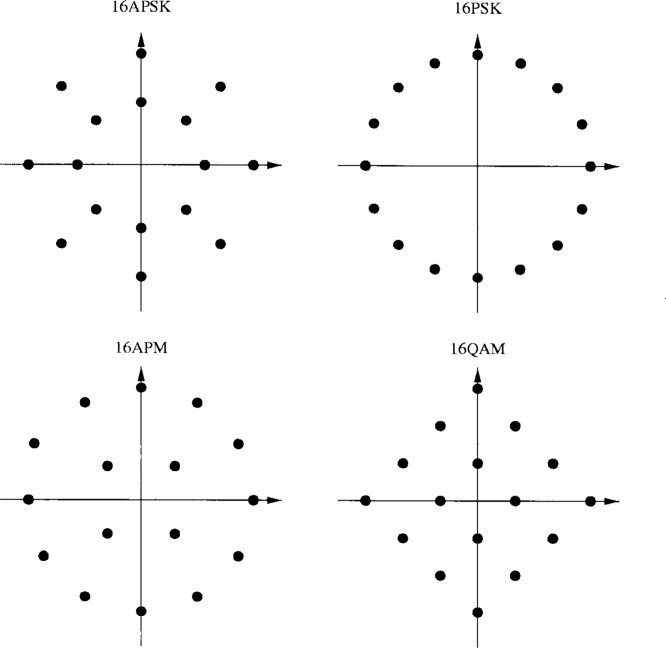
Signal constellations.

The performance of the modulation scheme depends on the Euclidean distance between the signal points, and in particular the minimum distance. The points should therefore be distributed as uniformly as possible over a circle with radius decided by the maximum output of the HPA. The positions of the signal points are however restricted as a too large peek-to-average power will contribute to increased cost pressure. When rotational invariance is required, that put another constraint on the constellation. In Sec. 4 different modulation techniques with 4 bits per symbol are considered. The four are 16PSK, 16APSK, 16APM and 16QAM (see Fig. 25). When different signal constellations are compared, the average energy per symbol is often normalized to 1. When the maximum signal level is limited by the HPA, it is more interesting to compare the performance with normalized maximum symbol energy.

The optimum ratio between the rings with 16APSK modulation for transmission over an AWGN channel is the one giving the same Euclidean distance between the points on the inner ring and between the two rings:

$$\beta_{\text{APSK}}=\frac{1-\sqrt{2-\sqrt{2}}}{\sqrt{2}-1}\approx 0.57.$$ (10)

With 16APM the optimum ring ratio is given by:

$$\beta_{\text{APM}}=\frac{4}{\sqrt{2}+\sqrt{6}+2\sqrt{6+\sqrt{3}}}\approx 0.42.$$ (11)

In Table 4 the squared Euclidean distances between the signal points are compared for the different constellations. The minimum distance is largest for the 16APM constellation, and smallest for the 16PSK constellation. It is however important to note that while the normalized average symbol energy is 1 for 16PSK, it is about 0.66, 0.56 and 0.86 for 16APSK (*β* = 0.57), 16QAM and 16APM (*β* = 0.42), respectively. The minimum Euclidean distances normalized by the average symbol energy are equal to 0.15, 0.29, 0.39 and 0.31 for 16PSK, 16APSK, 16 QAM and 16APM, respectively. Hence, 16 QAM gives the best performance in terms of BER as a function of ${\bar{E}}_{b}/N_{0}$.

**Fig. 26:**
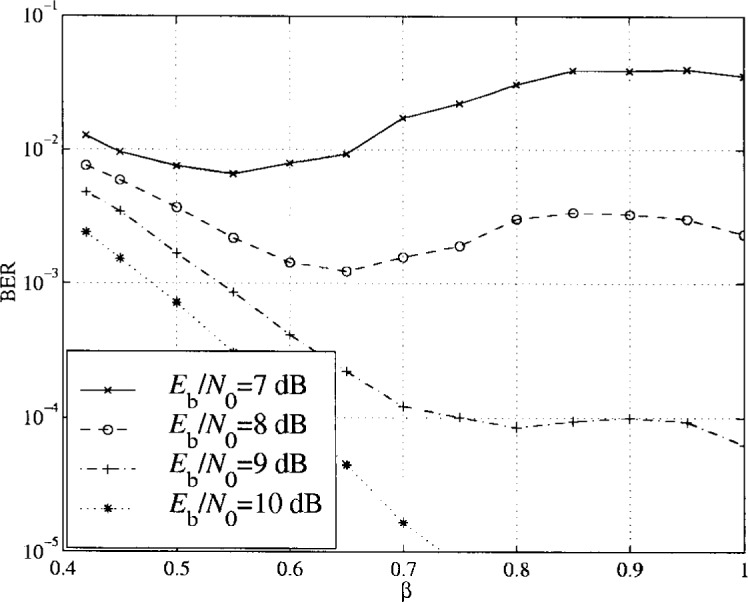
Simulated BER as a function of the ring ratio *β* and *E*_b_/*N*_0_ for 16APM.

**Fig. 27:**
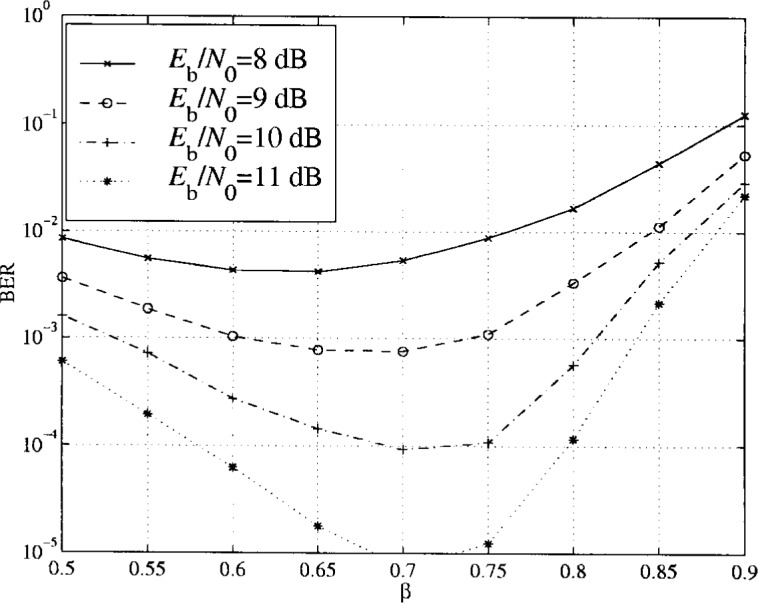
Simulated BER as a function of the ring ratio *β* and *E*_b_/*N*_0_ for 16APSK.

For PTCM coding, different bits have different error protection as only one out of *M* − 1 is coded. The optimum *β* value is therefore different than that for uncoded transmission. In the Figs. 26 and 27, simulation results of the BER with 16APM and 16APSK are shown for different signal-to-noise ratios and ring factors. For 16 APM, the positions of the points on the outer circle are modified so that the minimum distance between points on different rings are equal to the minimum distance between the points on the outer ring. Hence, with a ring factor of 1, the constellation corresponds to 16PSK. For low *E*_b_/*N*_0_ values, optimum *β* is close to 0.42, while for high *E*_b_/*N*_0_ values the optimum *β* value increases towards 1. Which ring ratio to choose then depends on the working point of the decoder. When Reed-Solomon outer coding is applied, for instance RS(204,188), a BER at the output of the PTCM decoder of 7 × 10^−4^ gives a BER at the output of the RS decoder of about 10^−9^ to −10^−11^, which is about the area for QEF transmission. In this area a ring factor of about 0.75 seems to be close to optimal. For 16APSK, the optimal ring ratio of about 0.7 seems to be optimal.

**Fig. 28:**
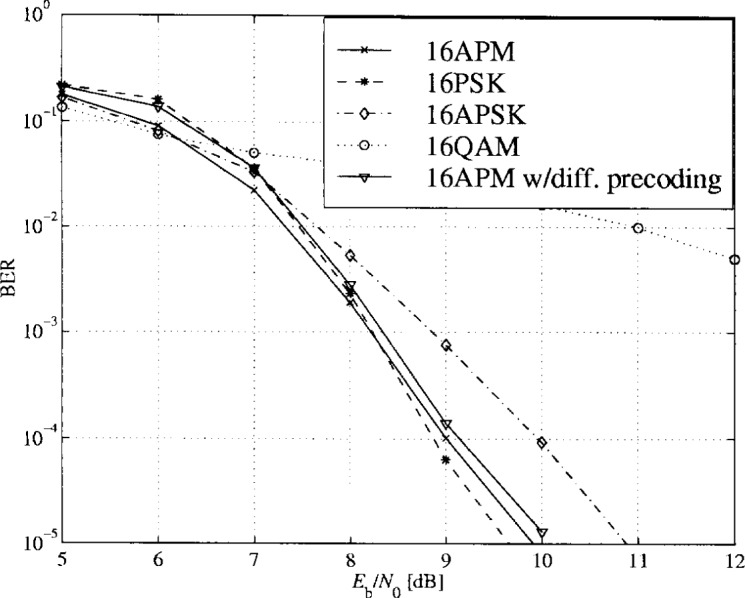
Simulated BER with PTCM encoding different modulations.

**Fig. 29:**
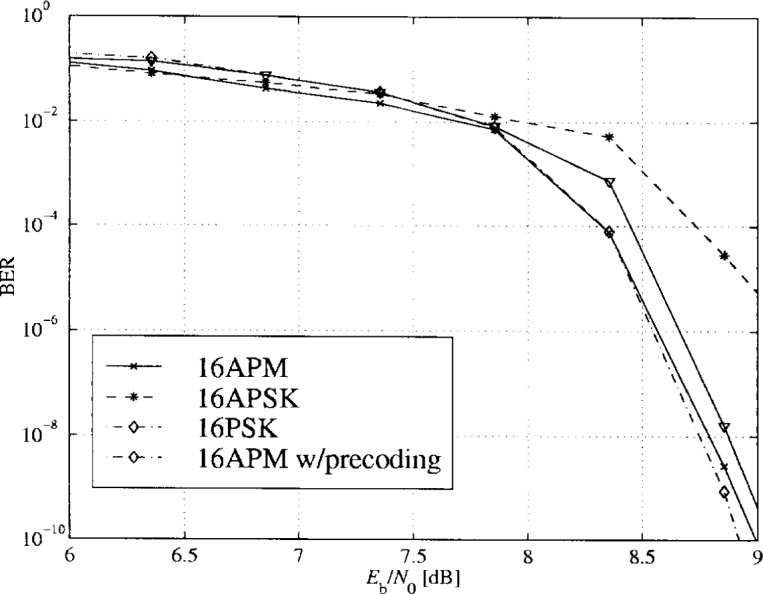
BER with RS(204,188) outer coding and PTCM inner coding and different modulation schemes. For 16APM the ring vector *β* is set to 0.75, and for 16APSK it is set to 0.7.

In Fig. 28, the simulated BER curves for PTCM with different 16-ary modulation schemes are depicted with normalized maximum signal level. 16APM and 16PSK give quite similar performance. The principal reason to choose 16APM is to obtain fully rotational invariance. The performance with RS outer coding is illustrated in Fig. 29. The BER at the output of the RS decoder is calculated as a function of the input BER using equations from [7]. Ideal interleaving is assumed.

### 4.4 Rotational Invariance

Most practical signal constellations have rotational symmetries. Rotations of the 16APM constellation about the origin by *i* × 90° leave the constellation unchanged for all integers *i*. For 16PSK the same is the case for rotations by *i* ×22.5°. The rotational symmetries introduce phase ambiguities in the receiver. If the receiver locks to a wrong carrier phase, and if the rotated sequence does not belong to the code, a long sequence of decoding errors will occur. Rotational invariant TCM (RI-TCM) schemes avoid this problem by ensuring that a rotated code sequence is always another code sequence, and that all rotations of a code sequence decode to the same information sequence. Thus, the receiver ignores the phase ambiguities rather than to try to resolve them.

Work on rotational invariance was first published in 1984 [14][15], where sufficient conditions for rotational invariance that apply on the trellis diagram were given. A number of RI-TCM codes, found either by ad hoc synthesis or by computer search over a restricted class of codes, have been proposed in the literature. The basic theory of rotational invariance is summarized in [16] and [17].

**Fig. 30:**
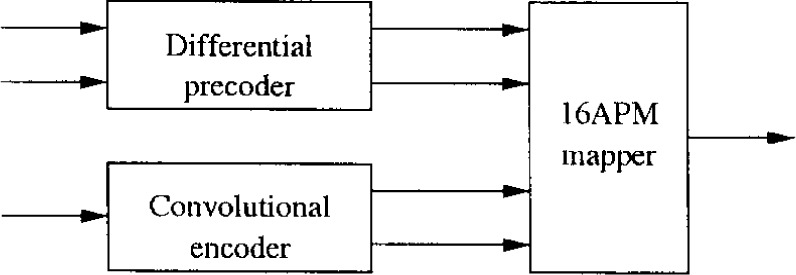
PTCM encoder with differential precoding.

For PTCM coding, rotational invariance can be obtained by the use of differential precoding. Phase ambiguities of multiples of 2π/2*^f^*, where *f* is the number of inputs to the differential precoder, are resolved using this form of differential encoding. Hence, to obtain 90° rotational invariance, it is enough to differentially encode two of the three encoder inputs, as illustrated in Fig. 30. Denoting the input and output of the differential precoder at time *t* = *kT* by *a_k_* and *b_k_*, respectively, *b_k_* is given by:

$$b_{k}=(a_{k}+b_{k-1})\mathrm{mod}\;2^{f}$$

where *a_k_*, *b_k_* ∈ {0,…, 2*^f^* − 1}. In Fig. 28 and Fig. 29, the simulated BER with differential precoding is included. The loss due to the differential precoding is about 0.15 dB.

The minimum rotation angle that leaves the 8PSK constellation unchanged is 45°. As maximum number of inputs to the precoder is two, fully rotational invariance can not be obtained. The constellation may be altered by moving every two signal points closer to the center, and thus making the constellation invariant for rotations of multiples of 90°. However, simulation results not included here indicate that the BER performance is severely degraded. A better solution is probably to use differential precoding to resolve phase ambiguities of multiples of 90°, and to resolve the remaining ambiguities by monitoring the growth of the metrics in the PTCM decoder.

The same is the case with QPSK modulation as for 8PSK. To obtain fully rotational invariance, differential precoding can resolve ambiguities of multiples of 180°, and by monitoring the metrics of convolutional decoder the remaining ambiguities can be resolved.

## 5. Channel Impairments and Approaches to Combat Them

### 5.1 Introduction

In order to design an architecture that enables LMDS systems to compete successfully with other broadband technologies, both in terms of price and performance, the modem must be matched to the RF part of the architecture and the propagation channel. The main channel impairments that need to be taken into account are signal attenuation, intermodulation products, phase noise and interference.

There are some important differences between the uplink and the downlink. The most important difference is the access scheme. Downstream information is basically broadcast, and data directed to one CPE is identified by an address embedded into the signal. For the uplink, some multiple access scheme is applied. The LMDS industry seems to have more or less discarded CDMA, and opts for TDMA/FDMA. TDMA is preferred if a large number of customers generate bursty traffic. FDMA is the best solution, for instance large companies, where a large number of users share one LMDS link. Another difference between up- and down-link is the antennas. Typically, the hub antenna has a beamwidth of 90°, while the CPE antenna has a beamwidth of 2° to 3°. Hence, CCI constitutes a bigger problem for the uplink than for the downlink. The transmitted power by the hub is higher than that of the CPE. The high power amplifiers (HPAs) in the hubs and the CPEs will therefore be different and having different characteristics. Typically, a solid state power amplifier (SSPA) [20] or a traveling wave tube (TWT) amplifier is used in the hub, while the CPE uses a GaAs MMIC amplifier. The non-linear distortion of the signal is consequently different in the downlink than in the uplink. Finally, to use low cost components in the CPE is crucial to reduce total CPE cost, while this criteria is not that important for the hub.

### 5.2 The Propagation Channel

The LMDS propagation channel has been studied through technical trials and propagation measurements conducted at several locations around the world. The characteristics of a real physical channel is of course very dependent on the environment of the actual system in hand. Parameters as frequency band and fixed antennas are however common for all LMDS systems, allowing some of the channel characteristics to be analyzed. In the design of any radio communication system a link budget analysis must be performed to established necessary transmitted power, antenna gains, modulation gains etc. to obtain a given level of performance, availability and maximum range. The free space loss together with other effects like clear air absorption and rain are briefly described below.

#### 5.2.1 Free Space Loss

The free space path loss is well known and can be expressed as a function of distance and wavelength:

$$L_{S}={\left(\frac{\lambda }{4\pi d}\right)}^{2}$$ (12)

or in dB:

$$L_{s,\text{dB}}=-92.45-20\;\log\;(fd)$$ (13)

where *f* is the frequency in gigahertz and *d* the distance in kilometers. For all realistic bandwidths, the difference in loss across the frequency band is negligible. For a distance of 2 km the free space loss at 30 GHz is about 128 dB, while for a distance of 8 km it is 140 dB.

#### 5.2.2 Rain Attenuation

Attenuation from precipitation will in many cases be the most significant threat to availability and QoS. It is necessary to include rain margins in the link budget in order to obtain required system availability. The attenuation depends on drop size, drop shape, rain rate and rain cross section. These factors depend on the climate, and rain region maps have been proposed by the ITU-R. The maps indicate the level of rain attenuation to be included in the link budget for a certain region and system availability. Examples for Dallas and Chicago show 3.95 dB/km and 2.28 dB/km loss for a system availability of 99.9 %, respectively [22].

The rain attenuation is related to the rain rate in an exponential manner:

$$\alpha =aR^{b}(\text{dB}/\text{km})$$ (14)

where the rain rate *R* measures in mm/h. The parameters *a* and *b* depend on the factors mentioned above, and their values for different climate regions and locations are available in the literature based on measurements and statistical calculations. As an example, in [27] the following values are given for the 30 GHz band with oblate spherical drops:

$$a_{h}=0.187,\;a_{v}=0.167,\;b_{h}=1.021,\;b_{v}=1.0$$

where the subscripts h and v denote horizontal and vertical polarization, respectively.

The dispersion due to rain is insignificant, i.e., no multipath is introduced by rain. However, when the drops are not spherical, the attenuation depends on the polarization of the signal. With the values cited above, the difference in attenuation affecting horizontal and vertical polarized waves is 0.3 dB/m, assuming a rain rate of 10 mm/h. Hence, rain induced depolarization may cause problems when polarization diversity is used to increase the capacity of the system.

#### 5.2.3 Clear Air Absorption

Molecular oxygen and water vapor lead to additional attenuation, which varies slowly with temperature, pressure and humidity. The water vapour spectrum has a weak absorption line at 22.235 GHz, and stronger lines at 183.3 GHz and above. The wings of these lines create a continuum also extending into the LMDS band [24]. The oxygen spectrum has several strong lines centered near 60 GHz, and more at 119 GHz and above. Table 5 illustrates the the effect of clair air absorption as a function of temperature and relative humidity [21]. The table indicates that this effects should be accounted for, especially in hot and humid areas.

#### 5.2.4 Vegetation

A large number of publications and reports deal with the effect of vegetation on microwave transmission. The attenuation per meter can be found for different types of vegetation and vegetation density. For typical LMDS links, both transmitter and receiver antennas will be placed relatively high above the ground. Tall trees may however obstruct the propagation path, leading to serious signal degradation. Measurements with coniferous and deciduous trees indicate that the loss is in the order of 1 dB/m to 2 dB/m for the first meters of foliage [21]. The loss per meter decreases for deeper foliages.

#### 5.2.5 Solid Structures and Terrain

Solid structures and terrain obstructing the line-of-sight will generally make reception impossible. Diffractions are practically non-existent in this frequency band, as the “shadow” becomes very sharp. Only in special cases where for instance a house roof is just tangent to the line-of-sight, diffraction occurs. The attenuation is large even if the receiving antenna is just 1 m to 2 m below the grazing ray (10 dB to 20 dB depending on the distance from the diffracting edge and the receiving antenna).

Reflections of surfaces may create problems in form of discrete multipath. This may happen if the antennas are located close to the ground level, or if structures like buildings are located close to the direct transmission path. High gain antennas with beam width in the order of (2° to 5°) suppresses multipath interference coming from other directions. The problem of discrete multipath can in most cases be avoided by careful placement of antennas.

Reflecting surfaces may also be used to create non line-of-sight transmission paths to cover areas not having a direct line-of-sight to the transmitter. The additive loss due to the reflection depends on the roughness of the surface, and on the incidence angle. A surface of brick or concrete may results in a loss of 7 dB to 15 dB [27]. Construction of glass will result in much smaller loss.

### 5.3 Distortion Due to the HPA

Different HPAs are needed for the hub and for the CPE. While the hub typically transmits over 1 W, the transmitting power of the CPE is typically much lower (~0.1 W). The HPA generates intermodulation products (IMP) due to nonlinearity. The nonlinearity has two effects on the transmission system. The first is spectral widening of the signal, which leads to out-of-band interference or adjacent channel interference (ACI) in a multichannel signal. The second effect is distorted signal components within the channel bandwidth. As the output of the pulse shaping filter is distorted, the matched filter in the receiver is no longer matched to the transmitted signal, and intersymbol interference (ISI) is introduced. Driving the HPA close to saturation impairs the nonlinear effects. A simple approach to avoid nonlinear distortion is to back-off the amplifier several decibels from the saturation region, keeping the signal level in the linear amplification range. Unfortunately, this results in lower output power, and less efficient operation of the HPA. Hence, there is a tradeoff between the power efficiency and linearity in the design of the system.

**Fig. 31:**
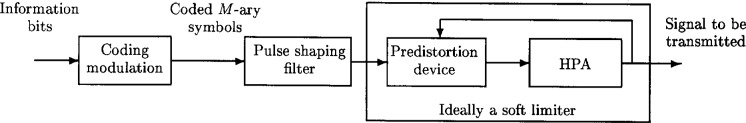
System model or evaluation effects of the non-linear HPA.

Figure 31 shows the system model used for evaluating the effects of the HPA. It contains a pulse shaping filter, a predistortion device and a HPA. These three components are described in the next subsections.

#### 5.3.1 Pulse Shaping Filter

**Fig. 32:**
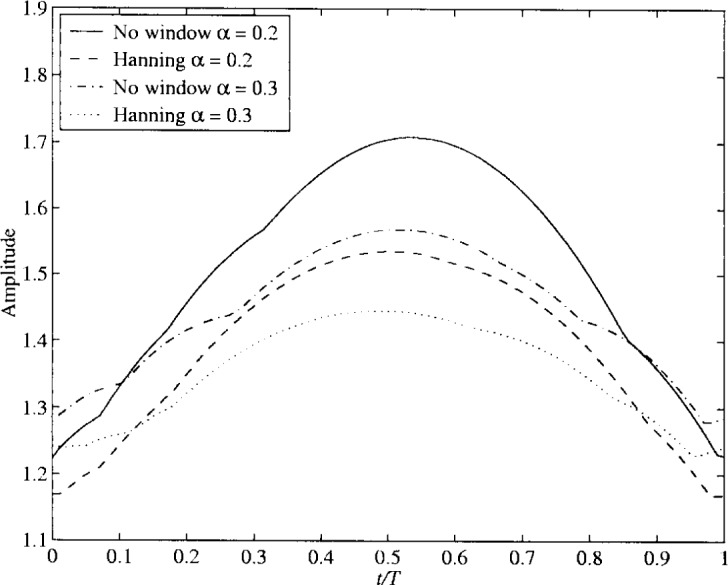
Maximum overshoot at the output of the square root Nyquist filter with and without Hanning window. The roll-off factor *α* is equal to 0.2 and 0.3, the filter input is 16APM symbols (*β* = 0.75), and the output is sampled 50 times per symbol.

In order to see the effect of the HPA on the system performance, it is necessary to know the characteristics of the input signal. The pulse shaping filter is a square root Nyquist filter with impulse response:

$$h_{\sqrt{\text{Nyq}}}=\frac{\left(4\alpha /T)\cos(\pi (1+\alpha )t/T)+\sin(\pi (1-\alpha )t/T\right)}{(\pi t/T)\left(1-{\left(4\alpha t/T\right)}^{2}\right)}$$ (15)

where *α* (0 ≤ *α* ≤ 1) is the cosine roll-off factor of the full Nyquist filter and *T* is the symbol duration. The impulse response is theoretically infinite. In the simulations, however, it is truncated with a total length is 2*LT*. The delay is then equal to *LT*. In modern communications with limited frequency resources, the roll-off factor is low, reducing the excess bandwidth. A low *α* has the disadvantage of relatively large overshoots when the signal goes from one level to another. This effect is illustrated in Fig. 32, where the maximum overshoot for the filter output is shown for *α* equal to 0.2 and 0.3. The curves are obtained by simulations, and the output of the filter was sampled with 50 samples per symbol. The input signal was 16APM symbols with a ring factor of 0.75. The pulse shaping filter function can by multiplied by a windowing function *h*_w_(*t*) to reduce the overshoots. The impulse response of the filter then becomes:

$$h(t)=\left\{\begin{array}{ll} h_{\sqrt{\text{Nyq}}}(t-LT)h_{w}(t) & 0\leq t\leq 2LT\\ 0 & \text{elsewhere} \end{array}\right\}.$$ (16)

A number of window functions may be used. In Fig. 32, simulation results using a Hanning window is included. The Hanning windowing function is given by:

$$h_{w}(t)=\frac{1}{2}\left(1-\cos\left(\frac{2\pi t}{LT}\right)\right),\;0\leq t\leq 2LT.$$ (17)

#### 5.3.2 The Traveling Wave Tube (TWT) Amplifier

In order to simulate the nonlinear effects, a commonly model of the TWT amplifier is applied. The input to the amplifier can be expressed by:

$$x(t)=A(t)\cos[\omega_{0}t+\theta (t)]$$ (18)

Non-linear distortions can be instantaneous or with memory. It is generally assumed that it is instantaneous, a hypothesis that holds when all the circuit time constants are much smaller than the signal envelope frequency [25]. In bandlimited transmission this will generally be the case [26]. The output is distorted both in amplitude and phase:

$$y(t)=G[A(t)]\cos\{\omega_{0}t+\theta (t)+\Theta [A(t)]\}$$ (19)

where *G* and *Θ* represent the amplitude and phase non-linearities, respectively, and are typically modeled as follows:

$$G(A)=\frac{\alpha_{1}A}{1+\beta_{1}A^{2}},\;\Theta (A)=\frac{\alpha_{2}A^{2}}{1+\beta_{2}A^{2}}$$ (20)

The parameters *α*_1_, *α*_2_, *β*_1_, and *β*_2_ are generally found by least-square fitting of the amplitude and phase characteristics of the TWT. In [27] the following values have been used:

$$\begin{array}{l} \alpha_{1}=1.0,\;\beta_{1}=0.25\\ \alpha_{2}=0.26,\;\beta_{2}=0.25. \end{array}$$

**Fig. 33:**
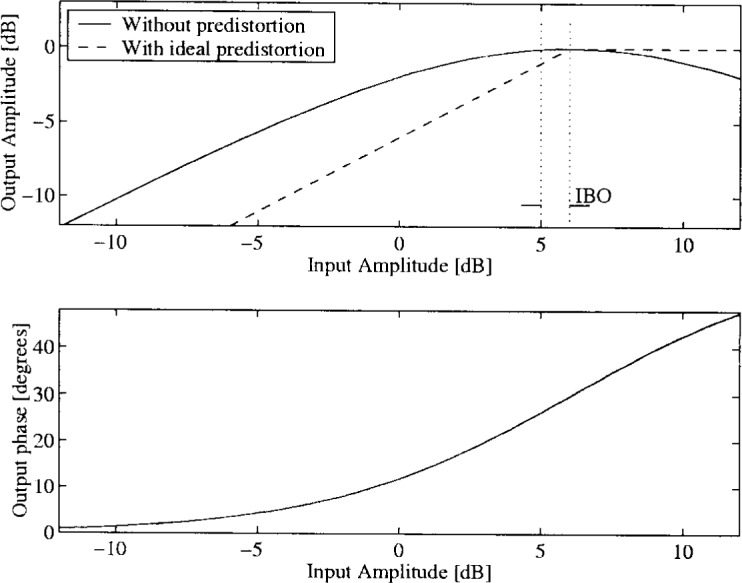
AM/AM and AM/PM characteristics for the HPA.

**Fig. 34:**
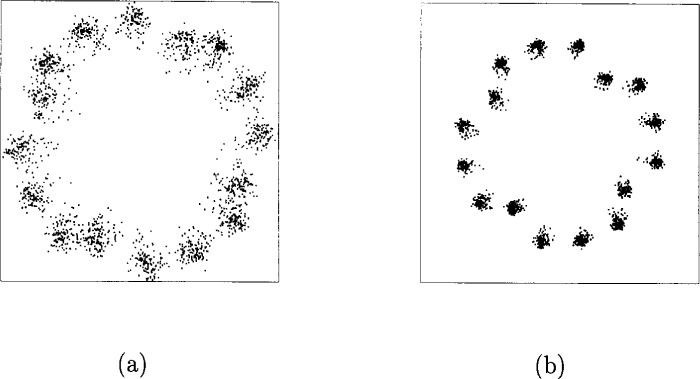
Scatter diagram the 16APM modulation (*β* = 0.7) with the HPA without predistortion. (a) IBO of 1 dB; (b) IBO of 6 dB.

In Fig. 33 the AM/AM and AM/PM curves are illustrated with these parameters. The scattering diagram using this TWT model is shown in Fig. 34. As expected, the received signal degrades, even with a large input back-off (IBO). With IBO as low as 6 dB, the degradation can be expected to be large enough to cause severe BER degradation.

**Fig. 35:**
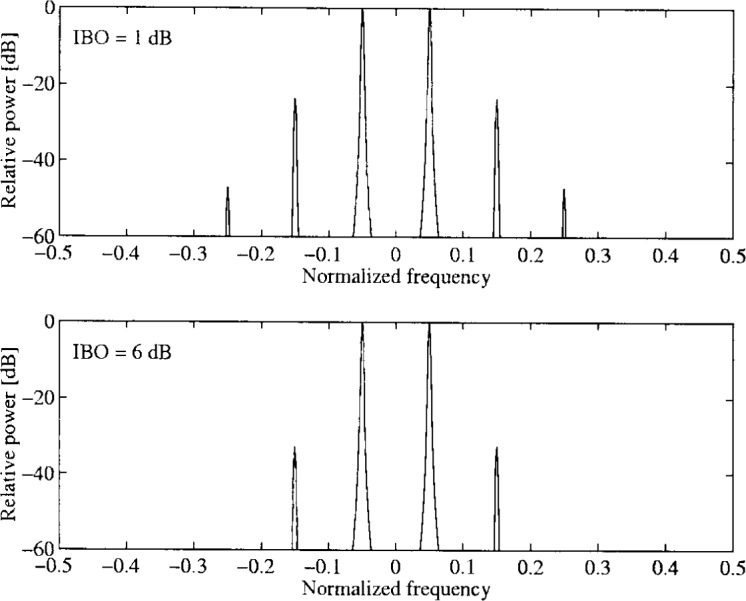
Intermodulation due to the nonlinear HPA. Input is a sinusoid with normalized frequency of 0.05.

In Fig. 35 the effect of spectral widening is illustrated. The input of the TWT is a sinus with a normalized frequency (with respect to the sampling frequency) of 0.05.

#### 5.3.3 Linearization and Predistortion

There are several approaches to compensate for the nonlinearity, including feed-forward linearization [28], Cartesian negative feedback linearization [29] and predistortion. The most widely used technique is predistortion [30]. The predistortion may be done digitally in baseband [31], or by analog processing [32]. The predistorting device should in any case be adaptive in order to compensate for the varying HPA characteristics due to temperature variations, aging, and other factors.

Digital predistortion may compensate for nonlinear systems with memory, i.e., the distortion introduced by the combination of the HPA nonlinearity and the memory of the filters. Analog predistortion only cancels out memoryless nonlinearities introduced by the HPA. The low price for silicon suggest that the digital approach may be the most cost-effective solution.

In digital predistortion the signal is digitized and pre-distorted using look-up tables [33]. The look-up tables can be configured in a number of ways, and be one or two dimensional. 1-D tables can be used since the distortion is essentially caused by amplitude variations. The tables may be based on polar representation or Cartesian representation of the signal. Digital predistortion can compensate for all distortion components, which is not the case for analog predistortion. Its main disadvantage is that it is bandwidth limited due to processing speed limitations. Essentially, it is an open loop technique, but it may be made adaptive by demodulating the output signal from the HPA and compare it with the required value [34].

With ideal predistortion, the cascade of the predistorting device and the HPA has no phase distortion, and the amplitude distortion may be modeled as a soft limiter, as illustrated in Fig. 33. Hence, clipping can not be avoided when input exceeds the saturation point. Some input back-off (IBO) from the saturation point is therefore necessary to avoid distortion.

**Fig. 36:**
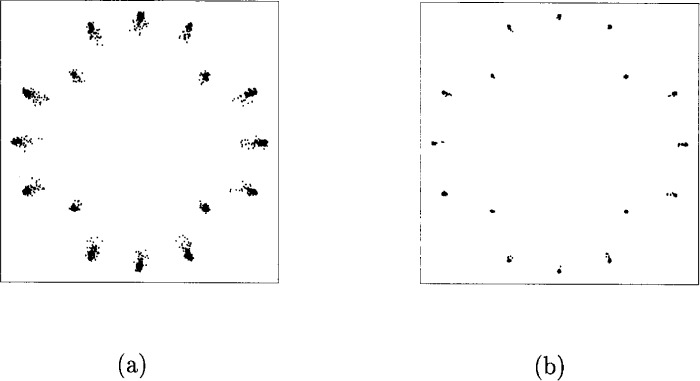
Scatter diagram with 16APM modulation (*β* = 0.7) and perfect linearization. *α* = 0.2. (a) IBO of 1 dB, (b) IBO of 3 dB.

**Fig. 37:**
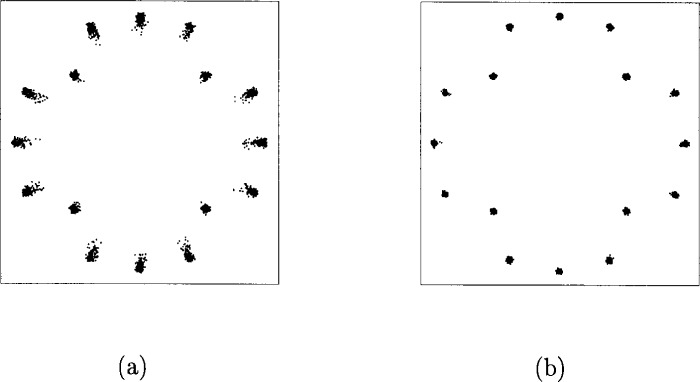
Scatter diagram with 16APM modulation (*β* = 0.7), Hanning window shaped pulse and ideal linearization. *α* = 0.2. (a) IBO of 1 dB, (b) IBO of 3 dB.

**Fig. 38:**
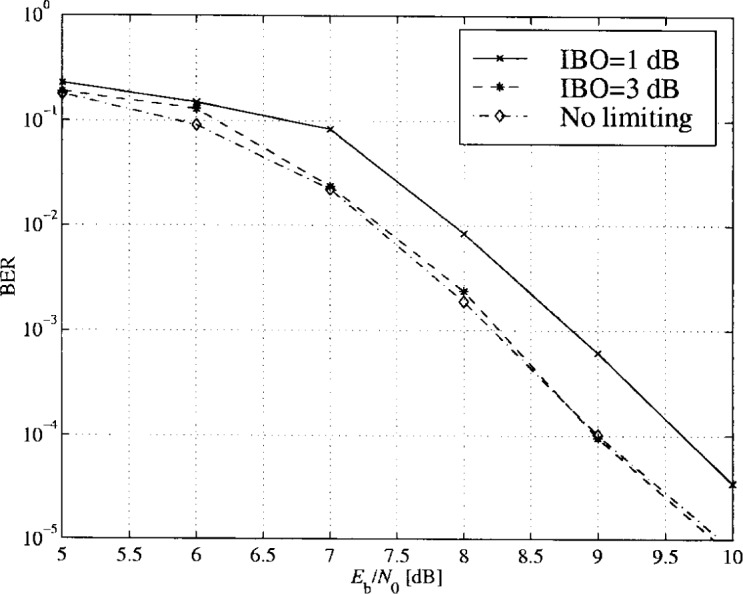
BER as a function of *E*_b_/*N*_0_ for ideal predistortion (soft limiting) with IBO equal to 1 dB; 3 dB and infinite.

In Fig. 36 the scatter diagram with perfect predistortion are illustrated with IBO equal to 1 dB and 3 dB. Compared to the scatter diagram obtained without predistortion, the quality of the signal is greatly improved. In Fig. 37 results using a Hanning windowed pulse shaping filter is included. The figure indicates that the loss is greater than the gain for this modulation scheme. The BER curves using perfect linearization (and no windowing function) are depicted in Fig. 38. With an IBO of 3 dB, the degradation is less than 0.1 dB for all BER of interest. With an IBO of 1 dB, however, the loss is about 0.6 dB to 0.7 dB.

### 5.4 Phase Noise and Carrier Phase Synchronization

The high transmission frequencies make phase noise a problem for LMDS systems. The main source of the phase noise is the millimeter wave local oscillator [22].

The receiver will lose the phase synchronization due to phase snaps or cycle slips. With non-rotationally invariant coding, the loss of sync results in a long burst of errors. With fully rotationally invariant PTCM coding, a phase snap will result in a short error burst at the output of the PTCM decoder. When the synchronization is reestablished, the decoder will quickly correct itself. The deinterleaver will spread the error burst out, and the outer RS decoder will correct the error bytes. Hence, the phase snap may have no impact on the BER. The increased insensitivity to loss of phase synchronization relaxes the constraints on the LNA and the VCO, permitting the use of lower cost components [23].

### 5.5 Effect of Interference

An important channel impairment is interference; interference generated by the system itself (intra-system interference) and interference from other communication systems (inter-system interference). The intra-system interference includes adjacent channel interference (ACI), intersymbol interference (ISI), and co-channel interference (CCI) due to frequency reuse. For U.S. LMDS systems, sources of inter-system interference include Ka-band satellite systems, stratospheric communications systems and other terrestrial (LMDS) systems. This section deals with the impact of interference on the system performance.

#### 5.5.1 ACI

As seen in the previous section, the non-linear HPA introduces spectral widening that puts some of the transmitted energy in adjacent channels, causing ACI. The transmitter power spectral mask defines maximum interference with adjacent channels. Table 6 is taken from the DAVIC 1.4 specifications, and shows the recommended downstream transmitter power spectral mask. By adjusting the guard band between the channels, the ACI can be made so small that it has no impact on the system performance.

#### 5.5.2 ISI

ISI is caused by multipath interference and non-ideal components. Results of a measurement campaign performed by the U.S. National Telecommunications and Information Administration (NTIA) [35] are given in Table 7. The transmitter and receiver antennas were mounted on 28 ft masts, and the propagation channels were partly obstructed by 30 ft to 50 ft tall trees. Three different channels were defined: good, moderate and bad. For all three channels, the delay spread is short. Compared to a symbol duration of 50 ns, corresponding to a channel bandwidth of 20 MHz, it is less than one tenth of a symbol duration. It will consequently not lead to significant ISI. Table 7 indicates, however, that the delay spread increases with the distance. Similar results have been reported in Ref. [36], using graphical information system (GIS) databases and a ray tracing software. For distances of several kilometers the delay spread may become so long that it has an impact on the system performance, and equalization becomes necessary. In order to adapt to different channel conditions, the equalizer must be adaptive.

**Fig. 39:**
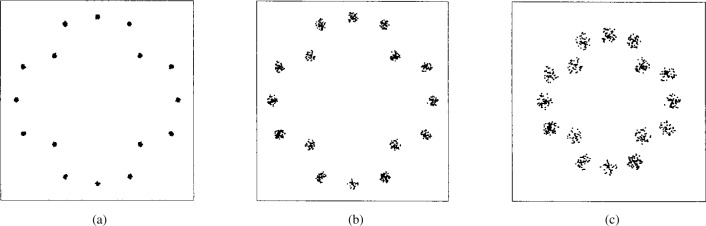
Scatter diagram with 16APM modulation (*β* = 0.7) with ISI. a) *N* = 2, *τ_n_*/*T* = (0, 0.1), *β_n_* = (1, 0.2), b) *N* = 3, *τ_n_*/*T* = (0, 0.1, 0.2), *β_n_* = (1, 0.4, 0.1), c) *N* = 4, *τ_n_*/*T* = (0, 0.1, 0.2, 0.3), *β_n_* = (1, 0.5, 0.3, 0.1).

Figure 39 illustrates examples of how the ISI may affect the signal using the tap delay mode:

$$h(t)=\sum_{n=1}^{N}\beta_{n}\delta (t-\tau_{n})e^{-j\omega_{c}\tau_{n}}$$ (21)

where *N* is the number of taps, *β_n_* is the tap gain, *τ_n_* is tap delay and *ω*_c_ is the carrier frequency. The diagrams show that the ISI degrades the signal even if the tap delays are well smaller than the symbol duration.

When the received signal is affected by ISI, the channel is frequency selective, i.e., the frequency response of the channel is not flat. Instead of using equalization to combat ISI, the channels can be divided into *N*_c_ sub-carriers. If *N*_c_ is large enough, the frequency response of each sub-carrier will be close to flat, and the ISI is eliminated. To avoid a decrease in system capacity, OFDM with its heavily overlapped sub-carriers should be used. Recently, the use of coded OFDM (C-OFDM) has been proposed for LMDS systems [36]. The channel state information (CSI) of each sub-channel is assumed to be known, which is reasonable as the channel is constant or very slowly varying and data flows in both directions. The implementation of this technique by means of the IFFT/FFT algorithm is quite efficient. OFDM is well suited for broadcasting of information, and not so well for multi-access communication. Another disadvantage with OFDM is large amplitude fluctuations that require a high degree of linearity in the HPA, and hence increasing the system complexity and cost. In [37] a combined OFDM/orthogonal CDMA (OCDMA) technique is proposed. The orthogonal codes are designed to minimize the nonlinear distortion. Both of these schemes are proposed for a scenario where a large number of residential users are offered a mix of TV-broadcasting and two-way data communications. This is not the scenario that is most likely to occur in North-America and in Western Europe. Furthermore, it is the market segment where CPE cost is most important, as the potential customers in most cases will have cable TV as an alternative. Taking that into consideration, techniques like C-OFDM are OFDM/OCDMA seem not to be the best choice.

#### 5.5.3 CCI

**Fig. 40:**
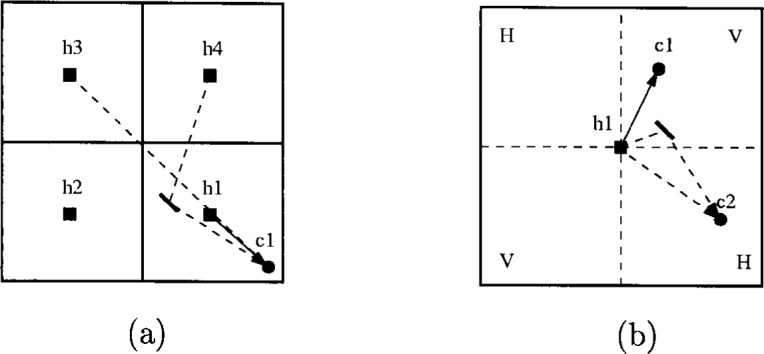
Example of (a) inter-cell and (b) intra-cell CCI.

Section 2.3 explains how cellularization and sectorization can be used to increase system capacity and availability. If each cell in a multi-cell LMDS network is assigned its own frequency band, the LMDS spectrum will be fragmented and the data rate offered to customers reduced. It is consequently desirable to reuse the same frequency band in several cells. The price to pay for this increase in capacity is inter-cell interference. Figure 40a illustrates a schematic example of inter-cell interference. There are four cells, for convenience drawn as squares, each with a hub in the center. There is one customer c1. The customer is located close to the extension of the line between the hubs h3 and h1. The signal from h3 will then interfere with the signal of interest from h1, if there is no obstruction in between. Signals from other hubs may also interfere if they are reflected by for instance tall buildings.

To further increase the capacity of the system, each cell can be divided into sectors, and the same frequency band reused in several sectors. Figure 40b illustrates a cell with four 90° sectors. The hub antenna has a beam width of 90°, and signals transmitted to c1 would ideally not reach c2. Practical antennas have a first sidelobe level of about 22 dB to 25 dB down from the main lobe, and some energy will leak out in the direction of c2. If the sectors use the same frequency band, the customer c2 will experience CCI. Reflections may also lead to interference. Sectorization is generally used in combination with polarization diversity, where adjacent sectors are assigned opposite polarization. This is illustrated in Fig. 40b, where H and V denote horizontal and vertical polarization, respectively. The polarization discrimination is about 18 dB to 20 dB [23].

**Fig. 41:**
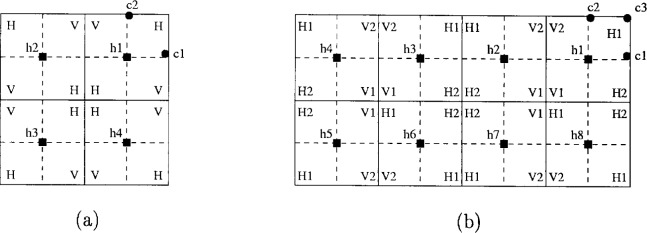
Frequency and polarization plan with (a) 1 frequency, 2 polarizations and 90° sectors, and (b) 2 frequencies, 2 polarizations and 90° sectors.

Both inter- and intra-cell interference can be reduced through a range of approaches. Spatial isolation reduces the inter-cell interference, but will also reduce the system availability. A combination of frequency planning and polarization reduces both inter- and intra-cell interference. The customer antenna has very narrow beamwidth, typically about 2° to 3°. The major part of inter-cell interference entering the receiver will therefore come from close to the same direction as the signal-of-interest. Assuming no obstructions, the interfering signal is then discriminated only by free space attenuation. The carrier to interference ratio (*C*/*I*) can in that case be calculated as:

$$C/I=20\log\frac{r_{1}}{r_{2}}$$ (22)

where *r*_1_ is the distance from the base station to the customer antenna, and *r*_2_ is the distance from the base station of another cell to the customer. In Fig. 41a, a polarization plan is illustrated with the same frequency band used by all cells. Each cell is divided into four 90° sectors. Neighboring sectors have opposite polarization. The locations c1 and c2 are the worst positions within the sector when it comes to carrier-to-interference ratio *C*/*I*. For c1, the signal of interest is transmitted by h1, and has horizontal polarization. The strongest interfering signals are transmitted from h1 with vertical polarization, and from h2 with horizontal and vertical polarization. Denoting the distance between hub 3 and the customers 1 and 2 as *r*, the carrier to interference ratio is given by:

$$C/I=\frac{Kr^{-2}}{vKr^{-2}+K{\left(3r\right)}^{-2}+vK{\left(3r\right)}^{-2}}=\frac{9}{10v+1}$$ (23)

where *K* = (λ/4π)^2^ and *v* is the polarization discrimination. With *v* = 18 dB, *C*/*I* becomes 8.9 dB.

Figure 41b illustrates the frequency and polarization plan with two frequency bands, two polarizations and 90° sectors. H*n* and V*n*, *n* = 1,2, denote horizontal and vertical polarization, using frequency band *n*. The customers at locations c1, c2, and c3 now experience the worst *C*/*I*. At c1, the signal of interest is transmitted from h1, uses frequency band 1 and has horizontal polarization. The main sources of CCI are the signals transmitted by h2 with vertical polarization, and from h3 with horizontal polarization. The *C*/*I* can then be expressed as:

$$C/I=\frac{Kr^{-2}}{Kv{\left(3r\right)}^{-2}+K{\left(5r\right)}^{-2}}=\frac{225}{9+25v}$$ (24)

With *v* = 18 dB, *C*/*I* equals 13.8 dB. With the use of two frequency bands instead of one, the worst case signal to interference ratio is then improved by 4.9 dB.

**Fig. 42:**
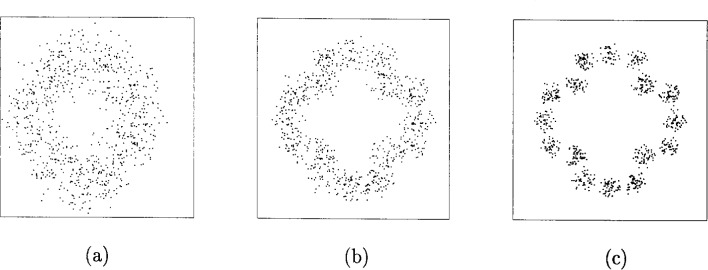
Scatter diagram with 16APM modulation (*β* = 0.75) with CCI. (a) *C*/*I* = 8.9 dB; (b) *C*/*I* = 13.8 dB; (c) *C*/*I* = 18 dB.

**Fig. 43:**
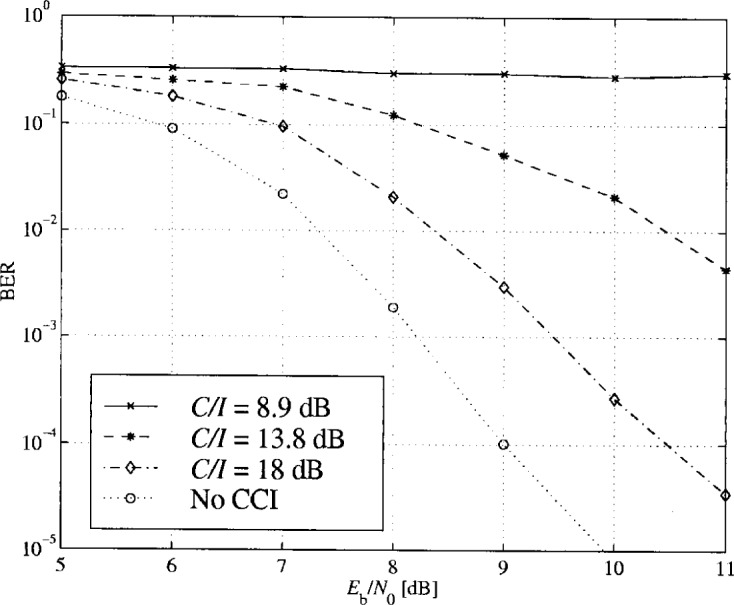
BER for PTCM with 16APM modulation (*β* = 0.7) with CCI.

The scatter diagram of the input signal of the decoder with CCI is illustrated in Fig. 42. The signal is severely degraded, even with relatively high *C*/*I* level. A *C*/*I* equal to 8.9 dB will, in most cases, make communication impossible. The impact of the CCI on the BER performances is illustrated in Fig. 43. Even with a *C*/*I* of 18 dB, the loss with respect to CCI-free transmission is about 1.5 dB. For *C*/*I* equal to 13.8 dB, the loss is about 4 dB to 5 dB and the BER curve reaches an error floor between 10^−3^ and 10^−4^. With a *C*/*I* of 8.9 dB, the error floor is above 10^−1^.

The average *C*/*I* level over the cell will be much higher than the worst case figures, the directive antennas, obstructions and more favorably locations will make sure of that. To be able to guarantee system availability of for instance 99.9 % or 99.99 % to all subscribers however, they must be taken into account.

#### 5.5.4 Inter-System Interference

In the United States, LMDS systems share the 27.5 GHz to 31.3 GHz band with satellite communications systems. The FCC has designated portions of this band to geostationary orbit (GSO) fixed satellite services (FSS), non-geostationary orbit (NGSO) FSS, and to feeder links for NGSO mobile satellite services (MSS). Of the three NGSO MSS systems Iridium, Globalstar and ICO, Iridium is the only one to have chosen to use Ka-band frequencies. It uses the frequency band 29.1 GHz to 29.3 GHz for uplink communications between gateways to satellites. Teledesic is a NGSO FSS system that will be using the band 28.6 GHz to 29.1 GHz for uplink communications when it starts operation sometime between the year 2003 and 2005. GSO FSS systems such as Astrolink and Spaceway, starting operation in 2001 or later, will use the band 29.25 GHz to 30 GHz for uplink communications. Of all these systems, Teledesic is probably the one that might cause problems for LMDS operators. Since Iridium gateways are fixed, it should be possible for LMDS operators to avoid interference from them with some system and cell planing. The GSO satellites are fixed with respect to the earth surface, so the same applies for them. If Teledesic, with its many NGSO satellites, gets a large number of subscribers in an area covered by an LMDS system, there might be interference problems. However, Teledesic will primarily provide backbone communications and not “last mile” services, so the number of ground terminals within the vicinity of the coverage area of a LMDS system should be small. The level of interference will depend on factors such as out-of-band transmission level for Teledesic terminals, and minimum elevation of satellites communicating with ground terminals.

Two conceptually different system solutions exist for stratospheric communication systems: manned or unmanned aircraft circling over the coverage area, and aerostats or balloons. The main exponents for the two solutions are the HALO system from Angel Technologies, and SkyStation, respectively. SkyStation will not constitute a source of interference for LMDS systems as it will use the 47 GHz to 48 GHz band. Angel Technologies, on the other hand, sees the use of the LMDS spectrum as one of several options for HALO. Such use of the LMDS spectrum could potentially create a problem due to the high altitude of the aircraft. It is likely, however, that if HALO is allowed to lease LMDS frequencies from an LMDS licensee, it will be under the conditions that its signals do not interfere with nearby LMDS networks.

Several LMDS networks may operate in the vicinity of each other, and potentially introduce interference. The U.S. LMDS spectrum is divided into a block A and a block B, and licenses for each block were auctioned separately. A portion of block A is squeezed between the two portions of block B, and out-of-band emission may increase the interference level. With a minimum of cooperation between LMDS operators however, mutual interference can be avoided.

## 6. Conclusions

The next 5 years will be crucial for the success of LMDS, at least in North-America and Western Europe. If LMDS operators have not by then obtained a fair share of the market, it will probably reside safely in the hands of wireline technologies such as xDSL, coaxial cable, and fiber.

A key issue in modem design is to reduce the cost of the RF units, which represent the main cost drivers for LMDS equipment. The modem must be designed with this in mind, and it must be matched to the LMDS channel.

Standards and specifications already developed for LMDS reflect an out-dated view that LMDS is a competitor to cable TV, primarily providing analog and digital television to residents. Even though the standards allow interactivity, they are not well adapted to the new world of integrated multimedia. New standards need to be developed to render LMDS systems competitive, and this work is under way through the IEEE 802 LAN/MAN Standards Committee.

Multicell LMDS systems tend to be interference limited due to frequency reuse. The potentially high interference level makes higher order modulation schemes such as 64QAM impractical. Careful cell planing and antenna placement is necessary to reduce the interference to a minimum and at the same time maximize availability throughout the coverage area.

## Figures and Tables

**Fig. 13:**
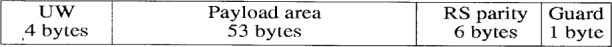
Frame structure for the upstream path.

**Fig. 15:**
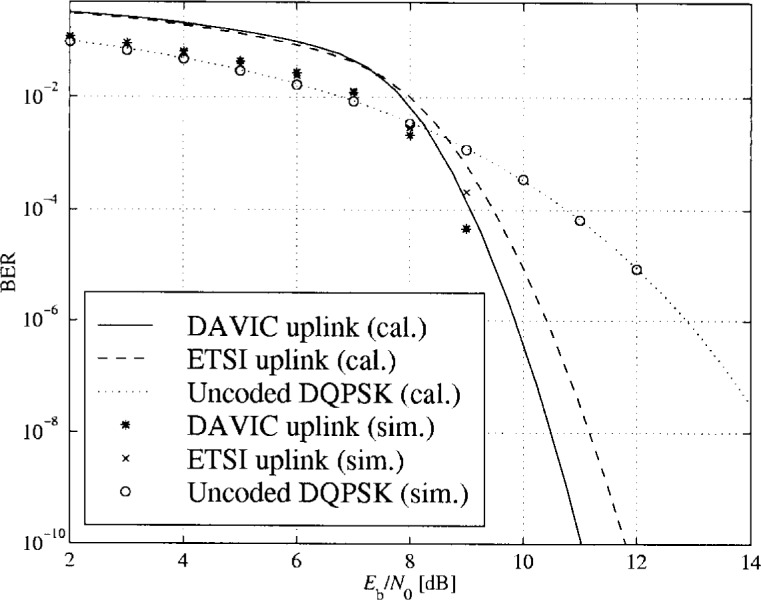
Performance for DAVIC and ETSI uplinks (DAVIC: RS(63,53) coding, DQPSK modulation, ETSI: RS(59,53) coding, DQPSK modulation).

**Table 1:** Punctured code definition

Code rates	2/3	3/4	5/6	7/8
*P*_1_	10	101	10101	1000101
*P*_2_	11	110	11010	1111010

**Table 2:** Summary of the ETSI and DAVIC specifications for the IF physical interface

	ETSI OOB down	ETSI IB down	ETSI up	DAVIC down	DAVIC up
Randomization	1 + *x*^5^ + *x*^6^	1 + *x*^14^ + *x*^15^	1 + *x*^5^ + *x*^6^	1 + *x*^14^ + *x*^15^	1 + *x*^5^ + *x*^6^
Outer coding	RS(55,53)	RS(204,188)	RS(59,53)	RS(204,188)	RS(63,53)
Convolutional interleaving	*I* = 5, *M* = 11	*I* = 12, *M* = 17	No interleaving	*I* = 12, *M* = 17	No interleaving
Convolutional (inner) coding	No inner code	Rate 1/2, 2/3, 3/4, 5/6, 7/8	No inner code	Rate 1/2, 2/3, 3/4, 5/6, 7/8	No inner code
Modulation	QPSK	QPSK	DQPSK	QPSK or 16QAM	DQPSK
Roll-off factor	0.3	0.35	0.3	0.2 or 0.35	0.3
IF frequency range	70 MHz to 130 MHz, 950 MHz to 2150 MHz	950 MHz to 2150 MHz	5 MHz to 305 MHz	950 MHz to 2050 MHz	400 MHz to 700 MHz
Channel spacing	2 MHz	Not specified	2 MHz	≥20 MHz, 1 MHz steps	*N ×* 100 kHz, *N* > 9
Frequency resolution	250 kHz	Not specified	50 kHz		
Channel bandwidth				20MHz to 40 MHz	Grade A: 1 MHz to 2.5 MHz Grade B: 1 MHz to 26 MHz
Symbol/bit rate per channel	3.088 Mbit/s	*k* × 8 kbps (guideline)	3.088 Mbit/s	14.81 MBd to 33.3 MBd, 8 Mbyte steps	Integer divisors of downstream rate (type 1 MBd)

**Table 3:** Free distance and weight structure for the convolutional code with constraint length 7 and generating polynomials 181 and 133.

*d*_free_	*w*_10_	*w*_11_	*w*_12_	*w*_13_	*w*_14_
10	36	0	211	0	1404

**Table 4:** Table over Eucledian distances between the points in the constellations

16PSK		16APSK		16QAM		16APM	

*N*	*d*^2^	*N*	*d*^2^	*N*	*d*^2^	*N*	*d*^2^
16	0.15	16	0.19	24	0.22	12	0.27
16	0.59	16	0.52	18	0.44	12	0.36
16	1.23	8	0.59	16	0.89	8	0.58
16	2.00	8	0.64	24	1.11	2	0.72
16	2.77	8	1.10	8	2.00	8	0.96
16	3.41	4	1.28	12	2.22	12	1.00
16	3.85	16	1.32	8	2.89	8	1.40
8	4.00	8	2.00	2	4.00	8	1.78
		16	2.12			20	2.00
		8	2.45			12	3.00
		8	3.41			12	3.73
		4	4.00			6	4.00

**Table 5:** Clear air absorption in dB/km at 28 GHz as a function of temperature and relative humidity [21]

Temp. (°C)	Rel. humidity 0 %	Rel. humidity 50 %	Rel. humidity 100 %
0	0.02	0.05	0.08
10	0.02	0.08	0.14
20	0.02	0.12	0.25
30	0.02	0.20	0.44
40	0.01	0.33	0.79

**Table 6:** Recommended downstream transmitter power spectral mask for DAVIC 1.4 specifications with 16QAM modulation [3] (*f*c: carrier frequency, *f*_N_: Nyquist frequency, *α*: roll-off factor)

$\left|\frac{f-f_{c}}{f_{N}}\right|$	16QAM Response [dB]	Tolerance [dB]
≤ 1 − *α*	0	±0.25
1	−3	±0.5
1+ *α*	<−24	
2 − *α*	<−36	
≥2	<−40	

**Table 7:** Tapped delay line model for measurements done in Northglenn, Colorado [35] (*β_n_*: tap gain, *τ_n_*: tap delay, *d*: distance, *A*: attenuation, *S*: delay spread (20 dB threshold), *L*_b_: transmission loss)

Quality	Tap #	*β_n_* (dB), 1 mW ref.	*τ_n_* (ns)	*d* (m)	*A* (dB)	*S* (ns)	*L*_b_ (dB)
Good	1	0	0	122	6.2	1.26	111.7
Moderate	1	0	0	309	32.2	1.60	145.9
	2	−13.7	5.3				
Bad	1	0	0	419	32.6	2.95	159.4
	2	−2.8	3.6				
	3	−16.2	15.3				

## Footnotes

1 Certain commercial equipment, instruments, or materials are identified in this paper to foster understanding. Such identification does not imply recommendation or endorsement by the National Institute of Standards and Technology, nor does it imply that the materials or equipment identified are necessarily the best available for the purpose.

## Glossary

ACI: Adjacent channel interference
APSK: Amplitude phase shift keying
ATM: Asynchronous Transfer Mode
AWDN: Additive white Gaussian noise
BC: Broadcast channel
BER: Bit error rate
CCI: Co-channel interference
CDMA: Coded division multiple access
CPE: Customer premisses equipment
CSI: Channel state information
DAVIC: Digital Audio-Video Council
DQPSK: Differential quadrature phase shift keying
DSL: Digital Subscriber Line
DVB: Digital video broadcasting
EP: ETSI project
ETSI: European Telecommunications Standard Institute
FDMA: Frequency division multiple access
FIFO: First in first out
FSS: Fixed satellite services
GIS: Graphical information system
GSO: Geostationary orbit
IB: Inband
IBO: Input back-off
IC: Interactive channel
IDU: Indoor unit
IF: Intermediate frequency
IMP: Intermodulation products
ISDN: Integrated Services Digital Network
ISI: Inter symbol interference
ITU: International Telecommunications Union
HPA: High power amplifier
LAN: Local area network
LFSR: Linear feedback shift register
LMCS: Local multipoint communication services
LMDS: Local multipoint distribution services
LNA: Low noise amplifier
MAN: Metropolitan area network
MAC: Media access control
MCNS: Multimedia cable network system
MMDS: Multichannel multipoint distribution services or Microwave multipoint distribution services
MPEG: Moving Picture Experts Group
MS: Microwave satellite
MSS: Mobile satellite services
MVDS: Multipoint video distribution services
NIST: National Institute of Standards and Technology
NMS: Network management system
NGSO: Non geostationary orbit
NTIA: National Telecommunications and Information Administration
N-WEST: National Wireless Electronic Systems Testbed
OAM: Operation, administration and maintenance
OFDM: Orthogonal frequency division multiplexing
OOB: Out of band
ODU: Outdoor unit
PCS: Personal communications services
PDH: Plesiochronous digital hierarchy
PHY: Physical layer
PLMN: Public land mobile network
PMP: Point-to-multipoint
PRBS: Pseudo random binary sequence
PSTN: Public switched telephone network
PTCM: Pragmatic trellis coded modulation
P^2^TCM: Pragmatic punctured trellis coded modulation
PTP: Point-to-point
QAM: Quadrature amplitude modulation
QEF: Quasi error free
QoS: Quality of service
QPSK: Quadrature phase shift keying
RF: Radio frequency
RI-TCM: Rotational invariant trellis coded modulation
RS: Reed-Solomon
SDH: Synchronous digital hierarchy
SL-ESF: Signaling link extended superframe
SOHO: Small office/home office
SONET: Synchronous optical network
SSPA: Solid-state power amplifier
TC: Technical committee
TCM: Trellis coded modulation
TCP/IP: Transmission control protocol/internet protocol
TDMA: Time division multiple access
TWT: Travelling wave tube
VCO: Voltage controlled oscillator
VLSI: Very large scale integration

## References

[1] Fuertes A (1998). Opportunities and Challenges Facing LMDS.

[2] McCabe T (1998). What lies Ahead for LMDS.

[3] (1998). DAVIC 1.3 Specification Part 8: Lower Layer Protocols and Physical Interfaces (Technical Specification), Revision 6.3.

[4] EN 301 199 V1.1.1 (1998-07), Digital Video Broadcasting (DVB) (1998). Interaction channel for Local Multi-point Distribution Systems (LMDS), Final Draft, European Standard.

[5] EN 300 421 V1.1.2 (1997-08), Digital Video Broadcasting (DVB) (1997). Framing structure, channel coding and modulation for 11/12 GHz satellite services, European Standard.

[6] Clark GC, Cain J Bib (1981). Error-Correction Coding for Digital Communications.

[7] Proakis JG (1995). Digital Communications.

[8] Cain JB, Clark GC, Geist JM (1979). Punctured convolutional codes of rate (*n* − 1)/*n* and simplified maximum likelihood decoding. IEEE Trans Inf Theory.

[9] Yasuda Y, Kashusi K, Harata Y (1984). High-rate punctured convolutional codes for soft decision Viterbi decoding. IEEE Trans Comm.

[10] Viterbi AJ, Zahavi E, Padovani P, Wolf JK (1989). A pragmatic approach to trellis-coded modulation. IEEE Commun Mag.

[11] Ungerboeck G (1982). Channel Coding with Multilevel/Phase Signals. IEEE Trans Inf Theory.

[12] Heller JA, Jacobs MI (1971). Viterbi decoding for satellite and space communications. IEEE Trans Commun Technol.

[13] (1987). QUALCOMM announces single-chip *K* = 7 Viterbi decoder device. IEEE Commun Mag.

[14] Wei LF (1984). Rotationally invariant convolutional channel coding with expanded signal space—Part I: 180°. IEEE J Sel Areas Commun.

[15] Wei LF (1984). Rotationally invariant convolutional channel coding with expanded signal space—Part II: Nonlinear codes. IEEE J Sel Areas Commun.

[16] Trott MD, Benedetto S, Garello R, Mondin M (1996). Rotational Invariance of Trellis Codes—Part I: Encoders and Precoders. IEEE Trans Inf Theory.

[17] Benedetto S, Garello R, Mondin M, Trott MD (1996). Rotational Invariance of Trellis Codes—Part II: Group Codes and Decoders. IEEE Trans Inf Theory.

[18] Lee CH, Chung BY, Lee SH Dynamic Modulation Scheme In Consideration of Cell Interference for LMDS.

[19] Wolf JK, Zehavi E (1995). *P*^2^ codes: Pragmatic Trellis Codes Utilizing Punctured Convolutional Codes. IEEE Commun Mag.

[20] Shu J, HWang T, Nguyen D, Pumares R, Chye P, Kenna P (1998). Ka-band 2 Watt Power SSPA for LMDS Application. 1998 IEEE MTT-S International Microwave Symposium Digest.

[21] Dalke RA, Hufford GA, Ketchum RL (1996). Radio Propagation Considerations for Local Multipoint Distribution Systems.

[22] Langston L, Marks R, Reese E Local Multipoint Distribution Tutorial.

[23] Liebetreu J BWA Standards—Modem Design View.

[24] Craig KH (1999). Propagation planning procedures for LMDS.

[25] Andreoli S, Banelli P, Marrocolo F, Massini C (1998). HPA Non Linear Distortions in DVB-T Systems, Simulation and Measurements.

[26] Saleh AAM (1981). Frequency-independent and frequency-dependent nonlinear models of TWT amplifiers. IEEE Trans Comm.

[27] Dalke RA, Hufford GA, Ketchum RL, Digital A (1997). Simulation Model for Local Mulitpoint and Multichannel Multi-point Distribution Services.

[28] Stewart RD, Tusubira FF (1988). Feedforward Linearisation of 950 MHz Amplifiers. IEE Proceedings.

[29] Johansson M, Mattsson T (1991). Transmitter Linearization using Cartesian Coordinate Negative Feedback for Linear TDMA Modulation.

[30] Park IS, Powers EJ (1997). An Adaptive Predistorter for High Power Amplifiers.

[31] Karam G, Sari H (1989). Analysis of Predistortion, Equalization, and ISI Cancellation Techniques in Digital Radio Systems with Nonlinear Transmit Amplifiers. IEEE Trans Commun.

[32] D’Andrea AN, Lottici V, Reggiannini R (1996). RF Power Amplifier Linearization Through Amplitude and Phase Predistortion. IEEE Trans Commun.

[33] Girard H, Feher K (1983). A new baseband linearizer for more efficient utilization of earth station amplifiers used for QPSK transmission. IEEE J Select Areas Commun.

[34] Faulkner M, Johansson M (1994). Adaptive Linearization Using Predistortion—Experimental Results. IEEE Trans Veh Technol.

[35] Papazian PB, Roadifer M, Hufford GA (1994). Initial Study of the local multipoint distribution system radio channel.

[36] Tralli V, Vaccari A, Verdone R, Andrisano O Adaptive C-OFDM System at 30 GHz for the Last Mile Wireless Broadband Access to Interactive Services.

[b37-j55hak] 37J. Li and M. Kavahrad, A Multiple Access Scheme for LMDS Based on OFDM and Sectored Antenna, to be published.

